# Evaluating the use of semi-structured crowdsourced data to quantify inequitable access to urban biodiversity: A case study with eBird

**DOI:** 10.1371/journal.pone.0277223

**Published:** 2022-11-09

**Authors:** Aaron M. Grade, Nathan W. Chan, Prashikdivya Gajbhiye, Deja J. Perkins, Paige S. Warren

**Affiliations:** 1 George Perkins Marsh Institute, Clark University, Worcester, Massachusetts, United States of America; 2 Department of Resource Economics, University of Massachusetts Amherst, Amherst, Massachusetts, United States of America; 3 Center for Geospatial Analytics, North Carolina State University, Raleigh, NC, United States of America; 4 Department of Environmental Conservation, University of Massachusetts, Amherst, Massachusetts, United States of America; University of California Los Angeles, UNITED STATES

## Abstract

Credibly estimating social-ecological relationships requires data with broad coverage and fine geographic resolutions that are not typically available from standard ecological surveys. Open and unstructured data from crowdsourced platforms offer an opportunity for collecting large quantities of user-submitted ecological data. However, the representativeness of the areas sampled by these data portals is not well known. We investigate how data availability in eBird, one of the largest and most popular crowdsourced science platforms, correlates with race and income of census tracts in two cities: Boston, MA and Phoenix, AZ. We find that checklist submissions vary greatly across census tracts, with similar patterns within both metropolitan regions. In particular, census tracts with high income and high proportions of white residents are most likely to be represented in the data in both cities, which indicates selection bias in eBird coverage. Our results illustrate the non-representativeness of eBird data, and they also raise deeper questions about the validity of statistical inferences regarding disparities that can be drawn from such datasets. We discuss these challenges and illustrate how sample selection problems in unstructured or semi-structured crowdsourced data can lead to spurious conclusions regarding the relationships between race, income, and access to urban bird biodiversity. While crowdsourced data are indispensable and complementary to more traditional approaches for collecting ecological data, we conclude that unstructured or semi-structured data may not be well-suited for all lines of inquiry, particularly those requiring consistent data coverage, and should thus be handled with appropriate care.

## 1. Introduction

Addressing the big challenges of the Anthropocene increasingly calls for big datasets [1–3]. Crowdsourced platforms have emerged as a powerful tool to gather large quantities of ecological data relatively inexpensively [3, 4]. Use of these datasets is particularly appealing for studying social-ecological patterns in cities, where spatial heterogeneity is high and complex patterns of land ownership pose challenges for accessing sampling locations [5, 6]. Critical reviews have identified a number of potential issues of bias, however, with the collection, use, and dissemination of information gathered by volunteers [7–9]. Many of the same social and technological barriers that have limited data gathering and information via traditional sampling methods (i.e. by trained experts) are likely to plague volunteer, user-gathered data as well [7]. This may yield socioeconomic, gender, and racial biases in who collects the data and uses the platforms [9–11], where users go to collect data [10, 12, 13], and consequently the representativeness of these datasets, particularly for historically marginalized communities [8, 13, 14]. The extent of sample selection bias in large, user-submitted data platforms remains largely unexplored (but see [13, 14]), but may pose particular challenges for open, semi-structured data platforms. This paper aims to assess how sample selection bias may affect studies using eBird, one of the largest crowdsourced platforms for biodiversity sampling [11], to analyze disparities in access to urban biodiversity.

Cities are spatially structured as a function of both historical and ongoing social and political processes that in most US cities yield a patchwork of racially and socioeconomically segregated communities [15–18]. A growing body of literature indicates that this spatial segregation is often associated with access to ecological amenities like open space, tree canopy cover, and biodiversity [18–21]. Non-representative sampling of urban spaces would therefore miss important variation in urban landscapes [13].

Crowdsourced contributory science (also known as citizen-science, hereafter, CS) data collection platforms decentralize data collection by crowdsourcing information from a broad base of users [22, 23]. eBird is among the most popular biodiversity-specific CS platforms. Its reach is global; eBird compiles information from users around the world for over 100 million bird sightings annually, with information on the location, time, and nature of bird encounters [24, 25]. This expansive geographic coverage makes it possible to assess biodiversity access in urban areas throughout the U.S. Given the widespread popularity of birds [26, 27], eBird represents a valuable resource for understanding not just the distribution of birds but of an important ecological amenity in urban spaces.

eBird relies on opportunistic semi-structured collection of data. Semi-structured methods allow users to gather data wherever and whenever they wish while collecting information on users’ observation process, such as effort, method, and location [22, 28, 29]. Collecting user process data distinguishes eBird from unstructured platforms like iNaturalist (iNaturalist.org), which is even more flexible and open to users’ submissions [29]. Structured programs, by contrast, set strict protocols. One example of structured programs is the Christmas Bird Count, which has set dates, sampling locations, and routes [30]. For brevity, we will refer to unstructured, semi-structured and opportunistic CS platforms as simply “SSCS platforms” hereafter, while recognizing the diversity of forms contributory science takes.

The decentralized nature of eBird presents both benefits and challenges. On the one hand, the SSCS model permits data collection at immense scale, and it can also generate public engagement with the scientific process. Platforms like eBird make possible analysis at a broader scope and finer scale than traditional ecological datasets, and can help pave the way for deeper inquiry into the relationship between access to biodiversity and human outcomes (and the disparities thereof). Yet these platforms, and SSCS initiatives more broadly, may also suffer from important shortcomings in terms of the representativeness of their data [31, 32]. This concern is particularly acute for investigations into diversity and socioeconomic disparities, as SSCS user bases and the places they report their data from within a city may be non-representative across racial and income groups. This type of selection bias may present two critical problems for within-city analyses using eBird data:

First, research using such data sources will not be able to shed light on impacts for underrepresented groups. Indeed, prior research has shown that CS users differ from the population as a whole along important demographic and locational dimensions [33, 34] and that the locations sampled by CS users are biased toward particular places [12, 13, 35]. Depending on the line of inquiry, these biases can jeopardize the generalizability of results.

Second, even deeper problems can arise for statistical inference, above and beyond the problem of generalizability. Because sampling sites are selected along variables of analytical interest (e.g., race and income composition of local communities), researchers may uncover spurious relationships between these variables and biodiversity. In particular, analyses of selected samples are prone to collider bias [36], a type of statistical bias that can lead to erroneous conclusions about the relationships between variables in a dataset. What is especially vexing is that one cannot predict, in general, whether collider bias leads to over- or underestimates of correlations in the data. In the presence of collider bias, variables that are truly uncorrelated may appear correlated, while variables that are truly correlated may appear uncorrelated [36, 37]. Both of these issues may pose a challenge to the utility of SSCS databases for conducting fine-scaled social-ecological assessments within cities, as SSCS databases are not set up within an experimental design framework and because of limitations to data coverage. Thus, sample selection presents a critical challenge for statistical inference in studies seeking to understand the relationship between community characteristics (like race and income) and access to urban biodiversity. Notably, this problem is not limited to the use of SSCS databases; it can arise in many settings with large, readily available datasets if researchers are inattentive to potential sample selection in how those datasets are constructed.

A few recent studies point to potential problems of sample selection bias in eBird and other SSCS platforms [12–14, 35]. Perkins [13] found a consistent pattern of geographic bias in the distribution of eBird sampling locations; neighborhoods in lower income groups were generally underrepresented among sites with checklists. In Buffalo, New York, another study found that sites with greater green space connectivity had greater numbers of submitted checklists, particularly sites near Lake Erie [35]. This study did not find a significant effect of socioeconomic factors on the distribution of checklists, but the sample size was relatively small since eBird checklists were reported for only 17% of the 50 block groups in the city [35], a fact which in itself suggests the potential for sample selection bias. A study of bird sightings posted via eBird, iNaturalist, and Flickr found that bird observations in Chicago occurred more often in open spaces than in residential areas, with high proportions of observations in recreation areas [12]. In addition, greater numbers of bird observations were posted for neighborhoods with higher median incomes, those with larger populations, and those located closer to Lake Michigan. While this latter study does not explicitly examine sample selection bias, the net outcome in all of these studies is that our information from SSCS data sources on the distribution of birds in the city may be biased and may have large gaps, particularly in those areas with little recreational space and/or low income.

We seek to fill three primary gaps with this study. First, prior work has not examined how the racial composition of an area correlates with its likelihood of being sampled for SSCS data collection. We test whether eBird checklists—an indication of the amount of available information about bird communities and biodiversity for a given area—are more prevalent in census tracts with a higher proportion of white residents and with higher median incomes, even after controlling for other relevant factors that may drive birding activity, like tract size and availability of green space. Note that our approach is distinct from related work that focuses on the racial and demographic composition of CS users [38, 39]; instead, we ask whether the spatial coverage of information available from SSCS datasets may be systematically biased toward neighborhoods of particular racial and socioeconomic composition. Second, much of the extant literature comprises case studies within single cities, leaving unclear whether results generalize across locales (but see [14]). We study two cities that are embedded in different ecoregions (temperate forest and arid desert, respectively) and have distinct urban geographies, finding similar results for both. Although our choice of study cities is not intended to be a comprehensive assessment of potential bias across the United States, the fact that two such distinct cities showed similar patterns of bias for a dataset as large and well-known as eBird suggests that sample selection is a wider spread problem when using SSCS datasets to assess social-ecological relationships. Lastly, we elucidate the broader implications of sample selection in SSCS databases. Not only does selection limit the generalizability of analyses of SSCS data, it may even compromise the validity of statistical inference in such studies due to collider bias. This problem is especially pronounced for studies using SSCS databases, like eBird, to link social and ecological variables, as the datasets themselves may be selected along social factors, leading to problems of collider bias. Our work is the first, to our knowledge, that pinpoints this challenge for using SSCS databases to study social-ecological relationships.

## 2. Methods

### 2.1 Study system

In this study we chose two United States (U.S.) cities for the analysis. Both cities are major metropolitan areas, but represent different bioclimatic and historic contexts, and thus represent much, but not all, of the variation in urban conditions across the U.S. Boston is one of the oldest cities in North America, and its metropolitan region is heterogeneous, dominated by high levels of wildland-urban interface [40] and aging post-industrial urban centers. The Phoenix metropolitan area experienced rapid urban growth beginning after World War II, and is dominated by extensive swaths of low-density residential lands that envelope large desert parks [41]. We conducted parallel, within-city analyses in both cities, demonstrating similar patterns of bias regarding what types of tracts are sampled in eBird.

### 2.2 Dataset compiling and filtering

**Fig 1:**
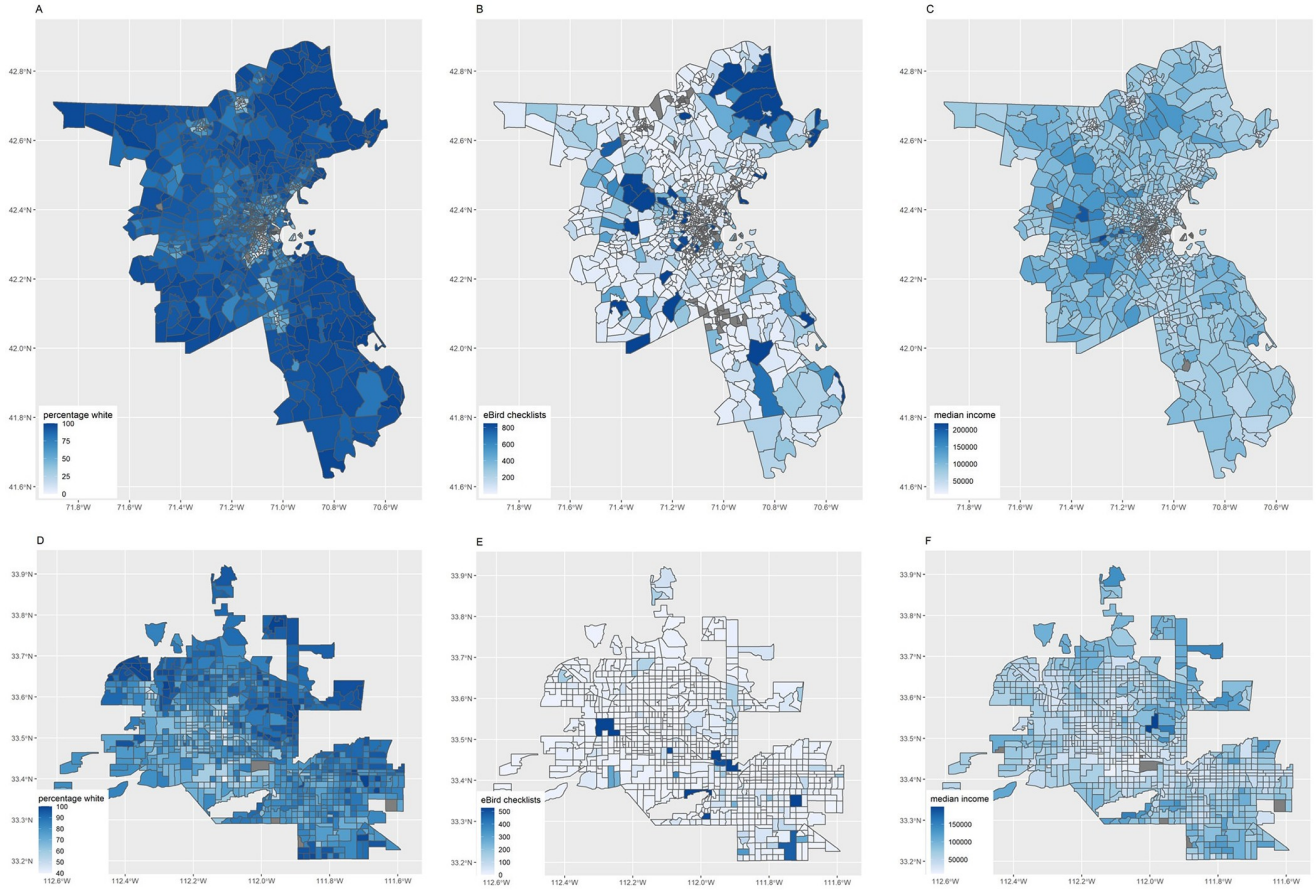
Study extent of census tracts analyzed for the Boston MSA (A,B,C) and Phoenix MSA (D,E,F). Darker blue indicates (A,D) higher percentage white residents, (B,E) more completed eBird checklists, and (C,F) higher median household incomes.

To examine the possibility of sample selection bias by the racial and income composition of areas sampled by eBird checklists, we compared land cover, socioeconomic, and demographic variables to numbers of eBird checklists in U.S. Census tracts within portions of the Boston, Massachusetts Metropolitan Statistical Area (BOS MSA) and the Phoenix, Arizona Metropolitan Statistical Area (PHX MSA). For spatial bounding purposes, we are using metropolitan statistical areas MSAs, but for ease of reading we are calling locations by their city names. The BOS MSA consisted of five counties within Massachusetts (Essex, Middlesex, Norfolk, Plymouth, and Suffolk Counties, **Fig 1A–1C**), and the PHX MSA consisted of only Maricopa County (**Fig 1D–1F**). To obtain socioeconomic and demographic variables, we downloaded and filtered U.S. Census and American Community survey datasets using the R package tidycensus (version 0.9.9.5; [42]). For all geographic data analysis, the datum used was GCS North American 1983 with an Albers Equal Area projection. We filtered the datasets to the counties indicated above, and summarized data at the census tract levels. We used five-year estimates (2006–2010) at the census tract level from the American Community survey to determine proportion of white residents within each census tract and median household income within a 12-month window in 2011 inflation-adjusted U.S. dollars. We used 2010 U.S. Census data to obtain overall population density (total people / ha) in each tract, since census tracts vary by total area.

We then examined the total number of eBird checklists collected between 1 Jan 2006 and 1 Jan 2016 [25] within each census tract and linked these to data from the 2010 U.S. Census [43] and American Community Survey for socioeconomic and demographic data [44]. The main object of analysis was thus the number of eBird checklists performed per tract, rather than the data collected within the checklists themselves. Checklists are a measure of effort and visitation per tract, which addresses the underlying question of sample selection bias by tract. We performed all data preparation and analysis using program R (version 3.6.2; [45]) and generated all plots using the R package ggplot2 (version 3.2.1; [46]). We downloaded and filtered eBird data using the R package auk (version 0.1.1; [47]; also see [23]). We filtered the dataset by clipping the data extent to all of the census tracts within the counties above, only complete checklists, the spatial and temporal extents indicated above, stationary and traveling eBird protocols only, distances traveled between 0–2.5 km, and checklist durations between 5–240 min [23].

To account for land cover and size of tracts, which were likely to be related to the total number of eBird checklists in a tract (e.g., due to birder activity), we quantified key tract-level land cover metrics using ArcMap (version 10.5; [48]). For BOS MSA, we included the proportion of green space in the tract by using the Protected and Recreational Open Space GIS layer available from MassGIS ([49]; https://www.mass.gov/orgs/massgis-bureau-of-geographic-information), as well as the total area of each tract (ha). Since the majority of open space in PHX MSA is not dominated by green vegetation, we included three separate measures of land cover: proportion of green space (combination of forest, emergent wetlands, golf courses, and parks), proportion of desert/scrub, and proportion of cropland, all of which we compiled from the National Land Cover Dataset 2011 GIS layer ([50]; https://www.mrlc.gov/data/nlcd-2011-land-cover-conus-0). We also included the total area of each tract (ha). For PHX MSA, we determined that many census tracts in Maricopa County were large uninhabited or very low-inhabited areas. To capture only the inhabited census tracts of interest in PHX MSA, we eliminated census tracts that were in the top 5% of land area (i.e., tracts ≥ 2,424 ha in area; tract size ranged from 32.6–345,074 ha).

### 2.3 Statistical analyses

Our goal was to study the relationship between sociodemographic variables and the prevalence of eBird checklists. We hypothesized that for each MSA, the number of eBird checklists at each census tract is related to metrics of race (proportion white) and income (median household income) of residents within the tract, even accounting for land area and areas available to birders (e.g., publicly available green and open space). We used the total number of eBird checklists for each census tract as the response variable in both BOS MSA and PHX MSA. For the predictor variables, we included proportion white and median household income as the hypothesized predictors, tract area, population density, and publicly available open space for covariate predictors. The publicly available open space specifically included proportion of green space for BOS MSA, and proportion of green space, proportion of desert, and proportion of cropland for PHX MSA. We check for excessive collinearity of predictors using the R package corrplot (v. 0.89). In the PHX MSA, median household income was highly correlated with total tract area and population density. Thus, we excluded total tract area and population density in the PHX MSA models. We found through exploratory analysis that many of the predictor variables were nonlinear. Thus, we decided to use generalized linear models with multistage model selection for analysis to choose the best fit probability distribution and link functions. We then validated the assumptions of normality of residuals post hoc.

Because of the possibility for multiple models with competing predictor structures, we used a maximum likelihood model selection and model averaging framework to assess the slope, significance, and effect size of variables [51–53]. To this end, for each MSA we conducted separate two-stage model selection procedures for the total number of eBird checklists in each city. We fit the GLMs using the R packages mgcv (version 1.8–31; [54]) and MASS (version 7.3–51.4; [55]; for models with negative binomial PDFs). For the first stage, we selected the best fit probability distribution (PDFs) and link functions of the GLMs by comparing hypothesized global models with different PDFs via Corrected Akaike’s Information Criterion (AICc; [56]) using the R package AICcmodavg (version 2.3.0; [57]). We considered any models within ΔAICc ≤ 2 to be equally likely [52]. Given the distributions of the eBird checklist count data, we tested the following PDFs: (1) gaussian (log + 1 transformation), (2) Poisson, (3) negative binomial, (4) inverse hyperbolic sine, (5) zero-inflated Poisson, and (6) zero-inflated negative binomial. The predictor structure for the BOS MSA hypothesized global model was:

*number of checklists = proportion of white residents × median household income + proportion of white residents + median household income + proportion of white residents^2^ + median household income^2^ + proportion of green tracts + proportion of green tracts^2^ + total tract area × population density + total tract area + population density + total tract area^2^ + population density^2^*

The predictor structure for the PHX MSA hypothesized global model was:

*number of checklists = proportion of white residents × median household income + proportion of white residents + median household income + proportion of white residents^2^ + median household income^2^ + proportion of green tracts + proportion of green tracts^2^ + proportion of cropland × proportion of desert + proportion of cropland + proportion of desert + proportion of cropland^2^ + proportion of desert^2^*

Once we selected the PDFs for each model set, we fit models using predictor variable structures from an *a priori* list of hypothesized and plausible models, which included additive and interactive linear terms as well as additive-only squared terms (as seen above in the global models; n = 220 models for each procedure for each MSA, see S1 Table for full candidate model set). We then assessed the models with the lowest AICc for spatial autocorrelation using the R packages sp (version 1.3–2; [58]) and gstat (version 2.0–6; [59]. We did not find evidence for spatial autocorrelation in the BOS MSA models, but we did find evidence for spatial autocorrelation at relatively short distances for models in the PHX MSA [60]. To account for spatial autocorrelation in PHX MSA, we reran the model selection procedure for PHX MSA and included latitude and longitude as additive terms in every model [61]. Once we fit all plausible models, we used the R package AICCmodavg to average the models and provide multimodal inference, using a 95% unconditional confidence interval to assess variable significance.

## 3. Results

After filtering the checklists, we assessed n = 122,299 eBird checklists in n = 740 census tracts for BOS MSA, and n = 17,779 eBird checklists in n = 861 census tracts for PHX MSA. In BOS MSA, the average census tract that we sampled had a median income of $79,102 and 81% of its residents are white (**Table 1**). The average census tract that we sampled in PHX MSA had a similar proportion of white residents (81%) and lower median income ($59,422). However, the variation in racial composition across census tracts is much wider in PHX MSA than BOS MSA; in PHX MSA, the tract with the lowest proportion of white residents is 4% white, while for BOS MSA this value is an order of magnitude large (40% white). We found no difference in the relationship between predictors and responses between total number of checklists versus number of stationary or traveling checklists, thus we present only the results of total number of checklists here. All models had a selected PDF structure of Gaussian with a log + 1 transformation (see **S1 Table**).

**Table 1:** Summary statistics by census tract for BOS and PHX MSA hypothesized predictor variables.

Predictor Variable	Minimum	Mean	Maximum
**BOS MSA**			
**Median income**	$13,545	$79,102	$218,667
**Proportion white**	0.04	0.81	1
**PHX MSA**			
**Median income**	$9,668	$59,422	$199,242
**Proportion white**	0.40	0.81	1

### 3.1 Boston MSA model averaging

**Fig 2:**
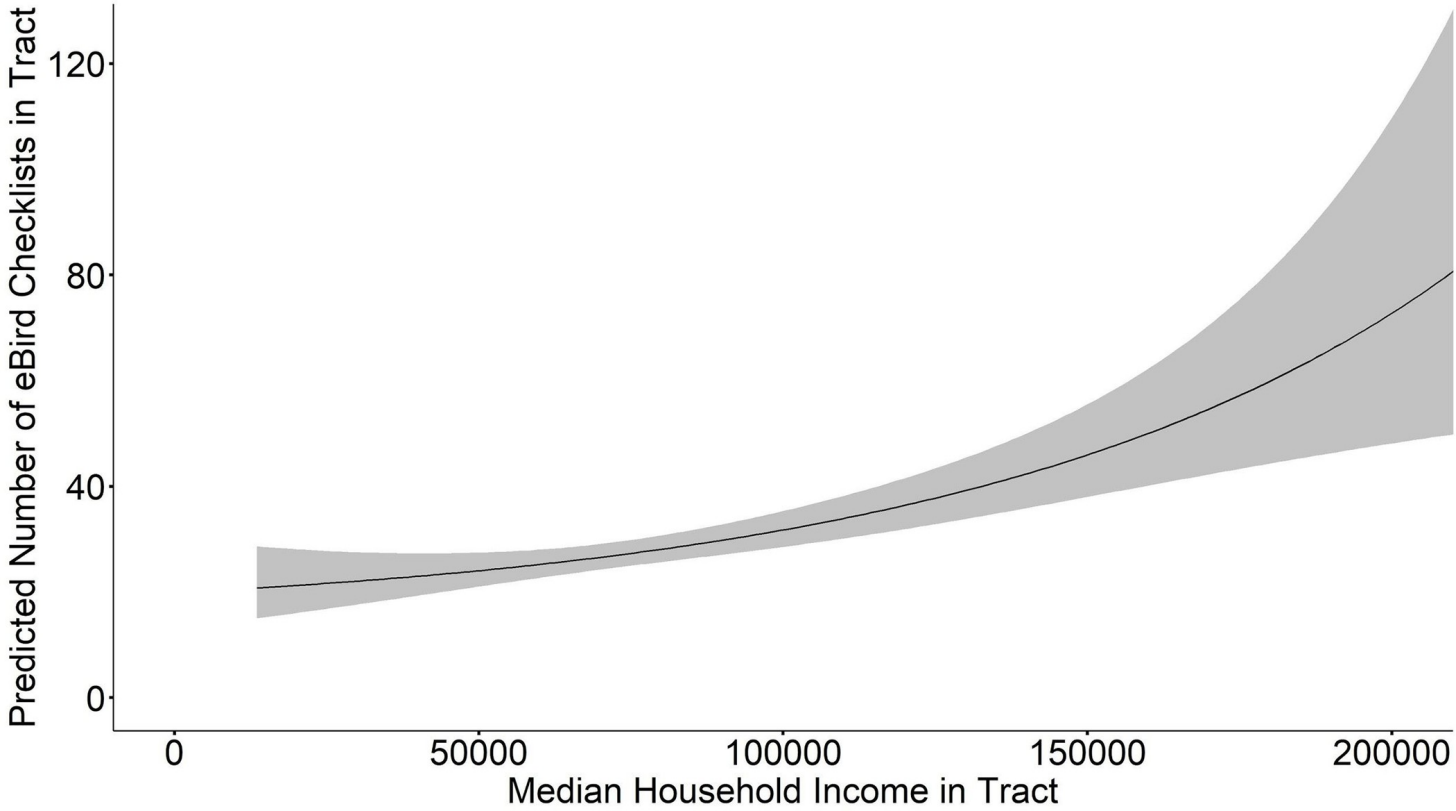
Model averaged relationship between median household income and predicted number of eBird checklists per tract in the Boston Metropolitan Statistical Area (BOS MSA). Gray ribbon indicates estimated standard error bounds.

**Fig 3:**
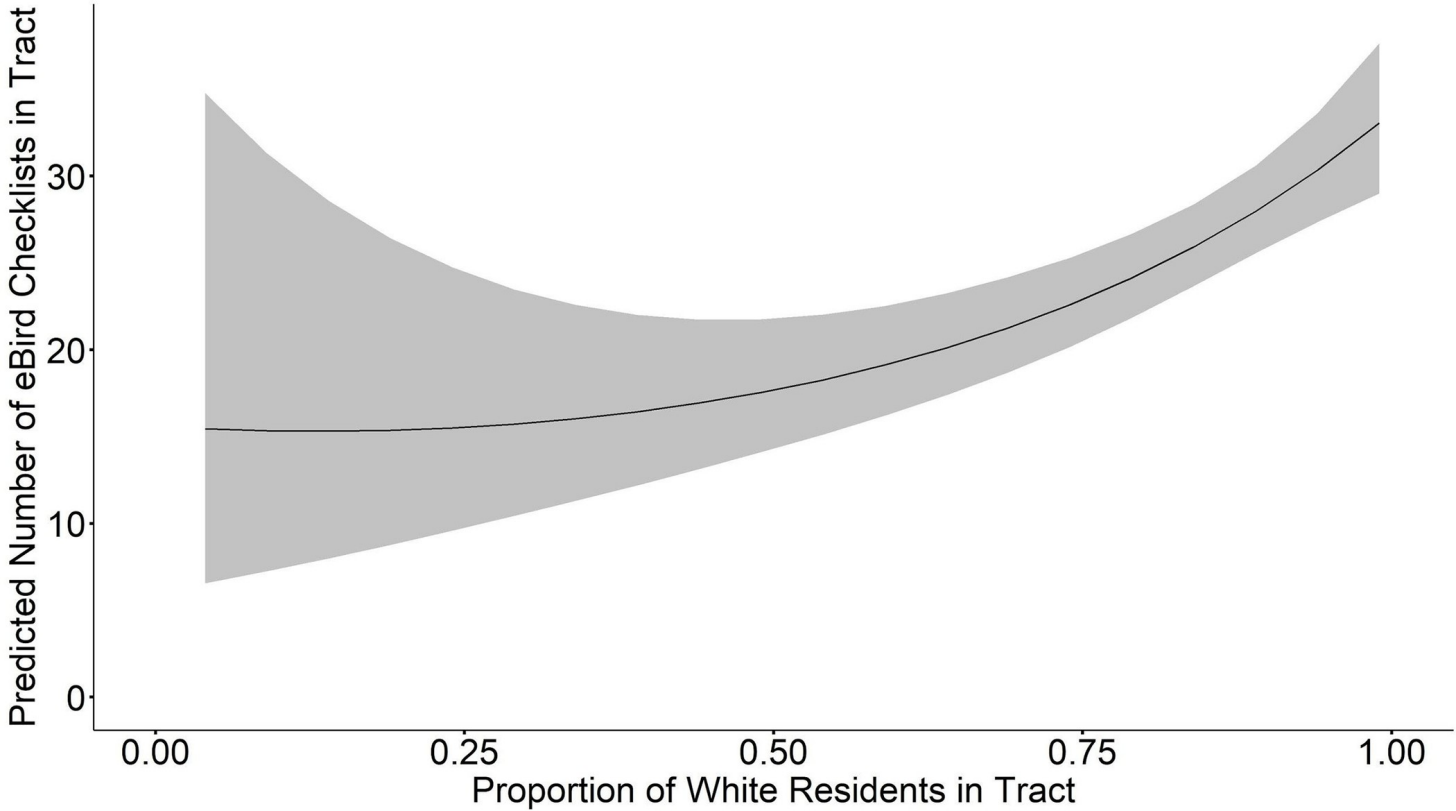
Model averaged relationship between proportion of white residents and predicted number of eBird checklists per tract in the Boston Metropolitan Statistical Area (BOS MSA). Gray ribbon indicates standard error.

When we averaged the model set for BOS MSA total eBird checklists, we found that median income, proportion white, proportion green space, total tract area, population density, median income × proportion white, and total area × population density were all significant at a 95% confidence level (**Table 2**). Proportion of green space × total area, and proportion of green space × population density were not significantly related to the total number of eBird checklists. Median household income and proportion of white residents were both included in all top models (ΔAICc < 2, w = 0.57); the R^2^ for these top models is 0.34–0.35. Median household income was positively related to the total number of checklists (β = 0.071, SE = 0.022, CI = 0.028, 0.114, **Fig 2**). Proportion of white residents in a tract was also positively related to the total number of checklists (β = 1.37, SE = 0.39, CI = 0.60, 2.13, **Fig 3**). These positive correlations between checklists, proportion of white residents, and median household income are robust and also hold for the top fit individual models.

**Table 2:** Model averaged parameters for BOS and PHX MSA total eBird checklists by predictor variables.

Predictor Variable	BOS MSA total eBird checklists	PHX MSA total eBird checklists
**Median income**	**0.071 (0.028, 0.114)**	**0.0602 (0.0146, 0.1058)**
**Median income** ^**2**^	0.0000 (0.0000, 0.0000)	0.0000 (0.0000, 0.0000)
**Proportion White**	**1.37 (0.60, 2.13)**	**2.308 (1.273, 3.342)**
**Proportion White** ^**2**^	1.8123 (-1.2768, 4.9015)	0.7006 (-4.558, 5.9591)
**Proportion green space**	**4.02 (3.04, 5.00)**	0.0002 (-0.0094, 0.0099)
**Proportion green space** ^**2**^	**-5.5737 (-10.455, -0.6923)**	0.0000 (-0.00002, 0.00004)
**Proportion desert**	N/A	**1.463 (0.729, 2.198)**
**Proportion desert** ^**2**^	N/A	-3.9602 (-9.0605, 1.14)
**Proportion cropland**	N/A	**1.5384 (0.4709, 2.6059)**
**Proportion cropland** ^**2**^	N/A	1.3416 (-1.5765, 4.2597)
**Total area**	**2.39 (1.30, 3.47)**	N/A
**Total area** ^**2**^	**-2, -0.5, -0.2)**	N/A
**Population density**	**-55 (-210, -89)**	N/A
**Population density** ^**2**^	**-50 (-9, -7)**	N/A
**Median income × proportion white**	**-0.305 (-0.602, -8.307)**	0.132 (-0.280, 0.543)
**Proportion green space × Total area**	4.04 (-10, 4)	N/A
**Proportion green space × population density**	-264 (-666, 139)	N/A
**Total area × population density**	**0.844 (0.105, 1.577)**	N/A
**Proportion green space × proportion desert**	N/A	**-0.052 (-0.103, -0.0007)**
**Proportion green space × proportion cropland**	N/A	0.248 (-0.402, 0.898)
**Proportion desert × proportion cropland**	N/A	5.019 (-4.864, 14.903)

Includes beta = model-averaged beta estimates with unconditional confidence intervals in parentheses. Income parameters are multiplied by 10,000. Bolded variables are significant (i.e., 95% CI does not overlap 0).

### 3.2 Phoenix MSA model averaging

**Fig 4:**
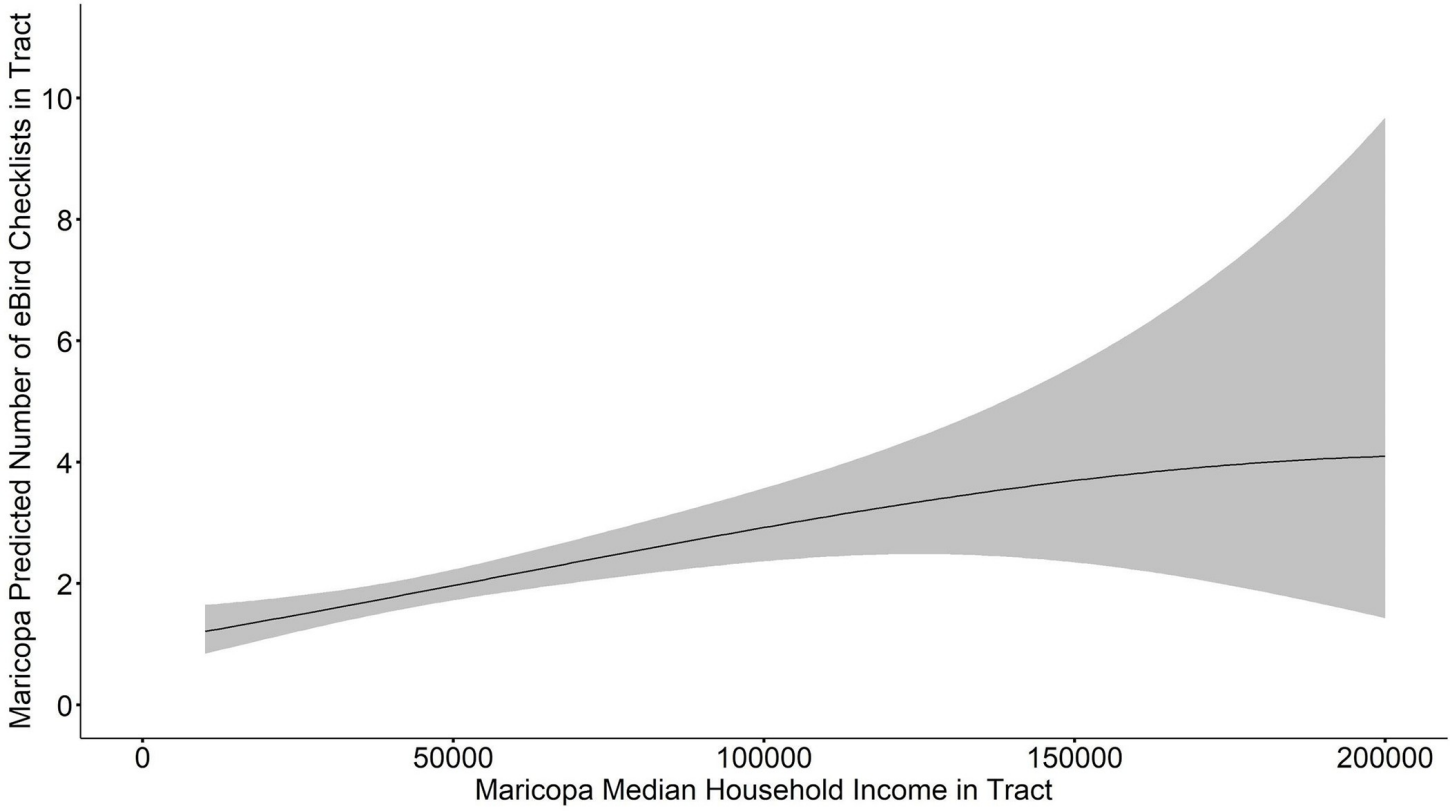
Model averaged relationship between median household income and predicted number of eBird checklists per tract in the Phoenix Metropolitan Statistical Area (PHX MSA). Gray ribbon indicates estimated standard error bounds.

**Fig 5:**
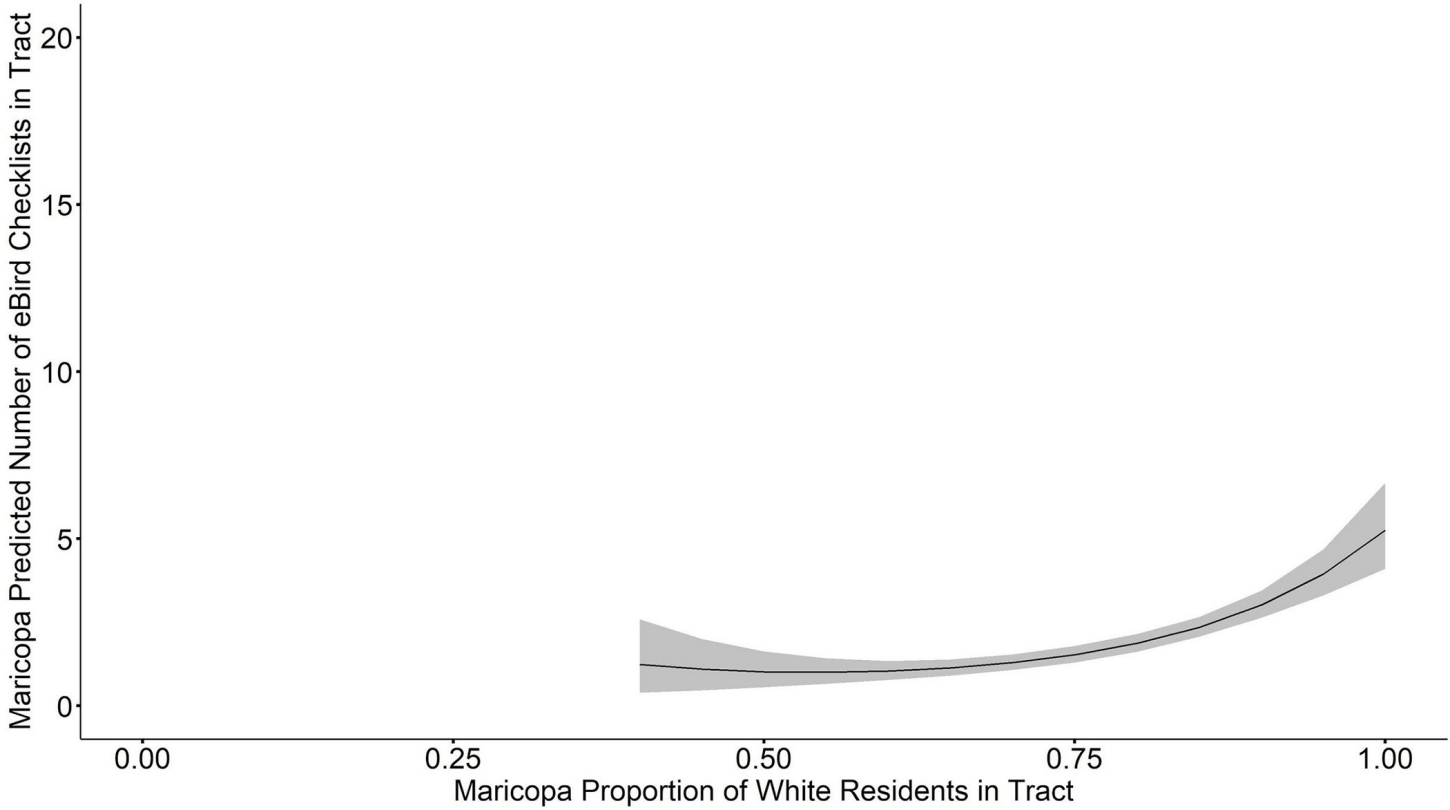
Model averaged relationship between proportion of white residents and predicted number of eBird checklists per tract in the Phoenix Metropolitan Statistical Area (PHX MSA). Gray ribbon indicates estimated standard error bounds.

When we averaged the model set for PHX MSA total eBird checklists, we found that median income, proportion white, proportion desert, proportion cropland, and proportion green space × proportion desert were all significant at a 95% confidence interval (**Table 2**). Proportion green space, median income × proportion white, proportion green space × proportion cropland, and proportion desert × proportion cropland were not significantly related to the total number of eBird checklists. Median household income and proportion of white residents were both included in all top models (ΔAICc < 2, w = 0.34, Table 2); the R^2^ for these top models is 0.13. Median household income was positively related to the total number of checklists (β = 0.060, SE = 0.023, CI = 0.015, 0.106, **Fig 4**). Proportion of white people in a tract was also positively related to the total number of checklists (β = 2.31, SE = 0.53, CI = 1.27, 3.34, **Fig 5**). These positive correlations between checklists, proportion of white residents, and median household income are robust and also hold for the top fit individual models.

## 4. Discussion

### 4.1 Sample selection and bias

We find strong evidence of sample selection in the locations where eBird activity is prevalent. This finding is in line with our hypothesis, and it suggests that census tracts are differentially likely to be surveyed for birds, and these differences are correlated with tract-level income and race. Especially notable is the prominence of tract-level income and proportion of white residents in predicting the number of available eBird checklists.

To our knowledge, only two other studies have directly examined racial and socioeconomic factors in relation to eBird checklist submission [13, 35]. A multi-city examination found significant differences in the distribution of eBird checklist submissions by neighborhood income level, with significant underrepresentation in the lower income areas [13]. In Buffalo, New York, socioeconomic factors were not a significant predictor of checklist submission [35]. However, this lack of relationship may have been due to the relatively small sample size (only 50 of 287 Census tracts had any eBird checklists submissions). The Buffalo study did find that eBird users tended to report checklists more often from places with greater green space connectivity and to avoid areas where active urban demolition projects have taken place [35]. Since green space coverage is greatest in wealthier areas of Buffalo, the biases in sample selection create a similar set of outcomes, i.e., that bird communities are under sampled in lower income portions of the city. Ongoing work by Ellis Soto and colleagues [14] finds biased sampling with respect to historic redlining in data from the Global Biodiversity Information Facility, a source that includes eBird records. Redlining refers to the racialized zones that the Home Owners Loan Corporation developed to guide lending practices in the early 20th century [16]. We note that none of these related studies explicitly examined biases associated with current racial composition of neighborhoods, though the Buffalo study and the redlining analysis accounted for covariates like the availability of green space [35]. In addition, our study encompassed the entire metropolitan region of our case cities, which extends far beyond the historically redlined portions of both cities. Together, these findings suggest that racial biases in sampling stem from ongoing social processes as well as historical ones, and that they merit further examination for eBird as well as for other SSCS data platforms.

The reasons for biased sampling in eBird and other SSCS datasets are likely to be multi-faceted. One possibility is that checklist submission might be driven by the availability of public green spaces where birders are attracted [12]. To the extent that green space or other natural features are less abundant in lower income neighborhoods or communities of color [19, 21, 62], the biases we detected might be attributable to disparities in preferred birding locations. However, as we have already indicated, accounting for percent green space in the tracts and population density did not eliminate race/income biases in the number of checklists submitted from census tracts in Boston or Phoenix (Table 2), nor did similar controls eliminate biased sampling aligned with redlining in a study of 195 cities [14]. There may, of course, be other factors associated with the accessibility of birding locations that we did not examine; a study in Sweden found factors like road density to be negatively associated with the sampling intensity across multiple taxa in a similar SSCS program [63]. Racism and classism may contribute to biased sampling if negative perceptions of lower income and non-white communities in cities leads to lower reporting from those areas [14, 18]. Likewise, a perception that certain birds are less interesting or worthy of reporting [64, 65] might contribute to under-sampling of areas perceived to be unlikely to support more prized species.

To the extent that the “where” of eBird sampling is related to who participates in eBird, racial and income biases in sampling may be a function of a lack of diversity among participants. eBird registrants are overwhelmingly white (94.8%; [11]). Analysis of participation in a water monitoring CS program, Illinois RiverWatch, provides some evidence for an association between the “who” and the “where” of SSCS sampling [10]; participants were disproportionately white and affluent and sites in areas of high environmental justice concern were under sampled. Broadening participation represents a key challenge for SSCS programs generally [11, 66]. Yet, a focus solely on the demographics of eBird contributors is likely an oversimplification. eBird participants do not solely or even predominantly submit checklists from their immediate home environments [12, 67]. Black birders and other birders of color may also experience significant barriers to accessing or using urban green spaces [68, 69], leading to further biases in spatial coverage of data availability. In addition, focusing only on rectifying sampling bias by calling for greater diversity in eBird participants could unfairly place the onus of gathering representative data on members of marginalized groups.

Critical reviews have examined the broad notion of volunteer-based geographic information systems (VGI). This term encompasses platforms that go beyond the goal of ecological sampling to include more general mapping tools (e.g. OpenStreetMaps—[9]), disaster management tools [8], and many other functions. Elwood [7] suggests that critical and feminist geography approaches provide frameworks for thinking about the sources of bias in VGI, and these can be extended to eBird and other environmental CS datasets. These approaches analyze the relationships between data and social and political power. For example, Elwood [7] notes that “unequal social and political relationships [may] influence spatial data access and sharing” and that the dynamics of inclusion and exclusion of spatially-explicit data may be influenced by “socially or politically-grounded motivations for volunteering or withholding.” In low income and communities of color, scientists have frequently engaged in extractive data collection [18, 70]. While SSCS data platforms like eBird have the potential to empower communities, there is also a risk that these platforms can contribute to further marginalization, depending on how these platforms are perceived and how the data is used [71, 72]. Further research is needed to assess how user perceptions of eBird might be influencing the spatial distribution of eBird checklist submissions.

### 4.2 The value of semi-structured crowdsourced data and future work

**Fig 6:**
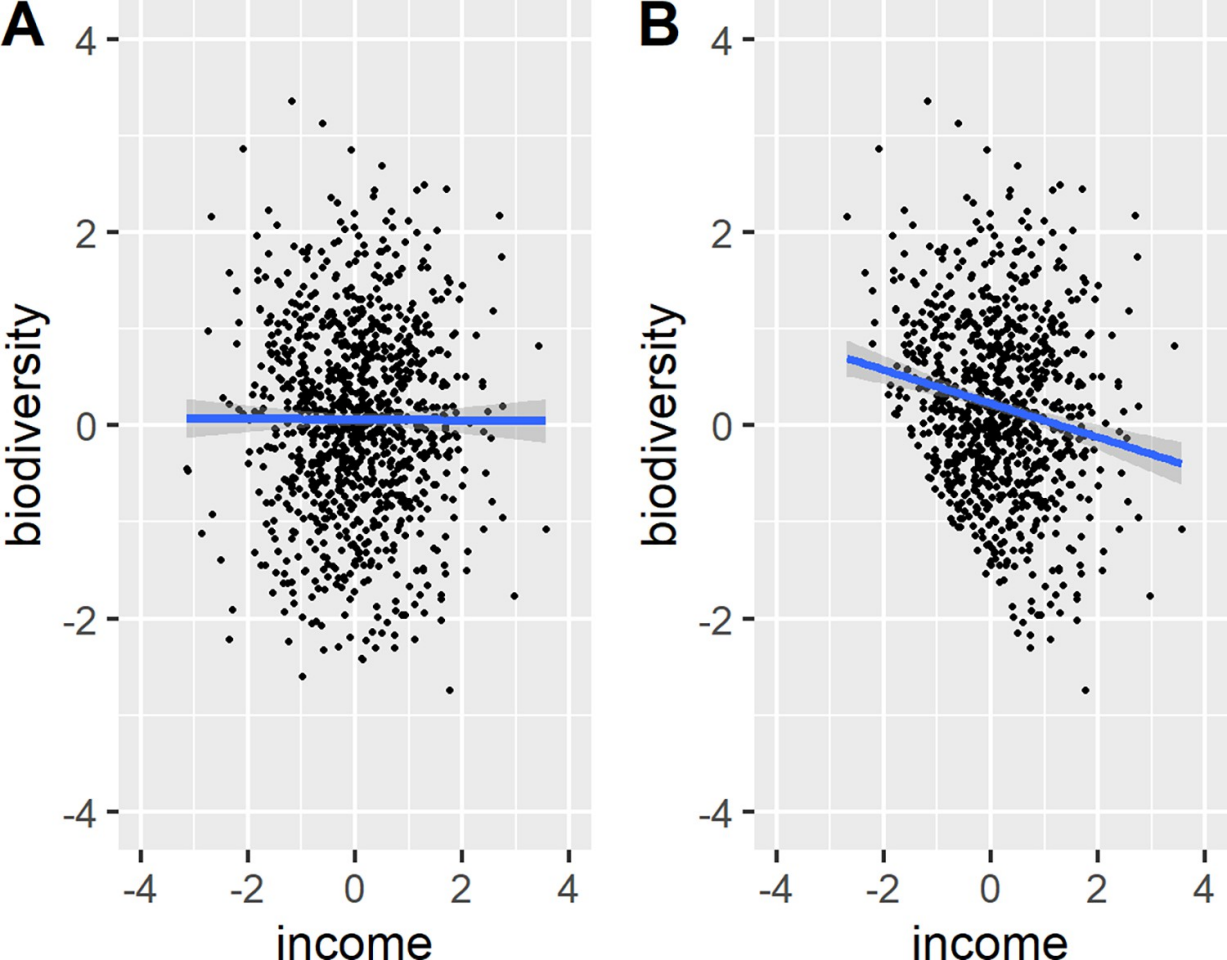
Simulated data to demonstrate the statistical bias that can occur with unbalanced sample selection. Panel A. Full sample with random, independent draws for biodiversity and income (β = -0.0042, SE = 0.031, p-value = 0.89). Panel B. Same sample with selection, where tracts with low income or low biodiversity are unobserved (β = -0.17, SE = 0.032, p-value < 0.001).

The preceding section suggests a need for caution when using SSCS data. In particular, unstructured or semi-structured data collection can lead to biased sampling along factors such as race, income, or geography. For this reason, SSCS data will not provide a reliable basis for assessing the relationship between these factors and access to biodiversity. Such analyses may suffer from collider bias, resulting in spurious associations. **Fig 6** provides an illustrative example (adapted from Cunningham [36]) of collider bias. Panel A shows the “true” underlying distribution of biodiversity and income for a hypothetical (simulated) set of tracts, while Panel B shows the distribution when tracts with low income or low biodiversity are unobserved due to selection. By construction, biodiversity and income are completely uncorrelated in the underlying simulation dataset (Panel A), but selection will lead the analyst to find a negative correlation between these two variables (Panel B). This figure clearly illustrates how selected sampling can lead to incorrect inferences about the relationship between variables of interest.

These problems are especially pronounced for studies examining within-city variations, where incomplete data coverage can pose challenges for valid statistical inference. Systematically sampled biodiversity data will be better suited for answering questions at such fine scales where SSCS platforms have data gaps. That being said, we should note that our primary empirical analysis focuses on the number of checklists, which is not a perfect proxy for biodiversity. Tracts with a low number of checklists may still have a sufficient number of checklists to describe biodiversity in that location, in which case concerns about collider bias will be mitigated. However, the problem of collider bias will remain a trenchant issue if many tracts have zero checklists. These tracts will appear to the researcher as though they have zero biodiversity or they may be systematically dropped from the analysis altogether—either of which would introduce bias into subsequent analyses.

In spite of these challenges, SSCS datasets remain important and powerful for many critical lines of inquiry. SSCS platforms like eBird have generated a wealth of data that are available at fine local levels and at unprecedented scales across the globe. These platforms provide unique and precious insights into social-ecological phenomena unfolding at regional and continental scales, such as migratory patterns [73, 74], changes in the geographic extent of plant and animal species [3, 75, 76], and human encounters with nature [67, 77]. In this light, we stress that SSCS data are indispensable and complementary to more traditional approaches for collecting ecological data. The central insight of this paper is that such unstructured or semi-structured data may not be well-suited for all lines of inquiry–particularly those requiring consistent data coverage–and should thus be handled with appropriate care. Against this backdrop, there are also opportunities to improve data collection to overcome some of the obstacles described above. SSCS platforms, like eBird, can create targeted campaigns to increase data coverage in tracts or areas with data gaps, thus ameliorating concerns about sample selection and collider bias. Moreover, expanding the user base of SSCS platforms, especially to underrepresented communities and areas, will improve data coverage by expanding the locations from which SSCS users originate and therefore where they are likely to report bird sightings.

Inclusivity, therefore, is crucial to CS efforts at multiple levels. Inclusivity is important to the outreach value of these efforts by expanding participation in and engagement with the scientific process [33, 66]. Yet, it is also essential to the rigor and validity of analyses that rely upon SSCS datasets. As we have demonstrated in this article, lack of inclusivity and incomplete data coverage can lead to critical errors in analyzing socio-ecological relationships, and these problems ultimately narrow the scope of questions that are answerable with SSCS data. We note, however, that these issues may also occur within many non-CS geospatial datasets across many fields [18, 20].

CS remains a powerful approach to connecting the public with science [13, 22, 66, 78]. Whose responsibility is it to address the gaps identified here and in other studies? Is it the managers of CS projects, or the academics who use the data? CS projects can have a variety of goals depending on who initiated the project and its primary purpose [22, 78, 79]. For example, a project whose aim is engagement and one whose aim is data collection are structured differently and engage people in different ways, yet are both categorized together as CS [66, 78, 80]. We urge ourselves and our colleagues in the field to do a better job of collaborating, and to bridge gaps between academia and project managers, in order to build CS datasets with greater reach, representativeness, and utility for scientific inquiry.

## Supporting information

S1 Table Models included in the model averaging for BOS MSA. (DOCX)

S2 Table Models included in the model averaging for PHX MSA. (DOCX)

S1 File (ZIP)
