# Supplementary material for: Evaluating the use of semi-structured crowdsourced data to quantify inequitable access to urban biodiversity: A case study with eBird
Source: PLoS One. 2022 Nov 9;17(11):e0277223. doi: 10.1371/journal.pone.0277223 (PMC9645630; doi:10.1371/journal.pone.0277223)
Supplement: S1 Table — (DOCX) [file pone.0277223.s001.docx]

**Table S1. Models included in the model averaging for BOS MSA.**Variable definitions: Prop_white = proportion of white residents, med_hh_income = median household income, tot_area_tracts = total area (ha), pop_dens_tracts = population density (residents / ha), and prop_green_tracts = proportion of tract that is public green space, measured at the tract level. ^2 refers to a squared term. K = number of parameters, AICc = Corrected Akaike Information Criteria, Delta AICc = Difference from lowest AICc value, AICc Wt. = AICc weight, Log Lik. = Log Likelihood, Cum. Wt. = Cumulative Weight.

| **Model Name** | **Predictor Variables** | **K** | **AICc** | **Delta AICc** | **AICc Wt.** | **Log Lik.** | **Cum. Wt.** |
| --- | --- | --- | --- | --- | --- | --- | --- |
| BOS_200 | perc_green_tracts + area_total_tracts x popu_dens_tracts + white_pct_acs_10 x med_hh_inc_acs_10 + white_pct_acs_10^2 + perc_green_tracts^2 + area_total_tracts^2 + popu_dens_tracts^2 | 13 | 2710.9 | 0 | 0.15 | -1342.2 | 0.15 |
| BOS_220 | perc_green_tracts + area_total_tracts x popu_dens_tracts + white_pct_acs_10 x med_hh_inc_acs_10 + white_pct_acs_10^2 + med_hh_inc_acs_10^2 + perc_green_tracts^2 + area_total_tracts^2 + popu_dens_tracts^2 | 14 | 2711.04 | 0.14 | 0.14 | -1341.23 | 0.28 |
| BOS_160 | perc_green_tracts + area_total_tracts x popu_dens_tracts + white_pct_acs_10 + med_hh_inc_acs_10 + perc_green_tracts^2 + area_total_tracts^2 + popu_dens_tracts^2 | 11 | 2711.13 | 0.24 | 0.13 | -1344.39 | 0.41 |
| BOS_210 | perc_green_tracts + area_total_tracts x popu_dens_tracts + white_pct_acs_10 x med_hh_inc_acs_10 + med_hh_inc_acs_10^2 + perc_green_tracts^2 + area_total_tracts^2 + popu_dens_tracts^2 | 13 | 2711.92 | 1.02 | 0.09 | -1342.71 | 0.5 |
| BOS_199 | perc_green_tracts + area_total_tracts + popu_dens_tracts + white_pct_acs_10 x med_hh_inc_acs_10 + white_pct_acs_10^2 + perc_green_tracts^2 + area_total_tracts^2 + popu_dens_tracts^2 | 12 | 2712.46 | 1.56 | 0.07 | -1344.02 | 0.57 |
| BOS_180 | perc_green_tracts + area_total_tracts x popu_dens_tracts + white_pct_acs_10 + med_hh_inc_acs_10 + med_hh_inc_acs_10^2 + perc_green_tracts^2 + area_total_tracts^2 + popu_dens_tracts^2 | 12 | 2712.92 | 2.02 | 0.05 | -1344.24 | 0.62 |
| BOS_170 | perc_green_tracts + area_total_tracts x popu_dens_tracts + white_pct_acs_10 + med_hh_inc_acs_10 + white_pct_acs_10^2 + perc_green_tracts^2 + area_total_tracts^2 + popu_dens_tracts^2 | 12 | 2713.03 | 2.13 | 0.05 | -1344.3 | 0.67 |
| BOS_159 | perc_green_tracts + area_total_tracts + popu_dens_tracts + white_pct_acs_10 + med_hh_inc_acs_10 + perc_green_tracts^2 + area_total_tracts^2 + popu_dens_tracts^2 | 10 | 2713.13 | 2.24 | 0.05 | -1346.42 | 0.72 |
| BOS_219 | perc_green_tracts + area_total_tracts + popu_dens_tracts + white_pct_acs_10 x med_hh_inc_acs_10 + white_pct_acs_10^2 + med_hh_inc_acs_10^2 + perc_green_tracts^2 + area_total_tracts^2 + popu_dens_tracts^2 | 13 | 2713.18 | 2.28 | 0.05 | -1343.34 | 0.76 |
| BOS_209 | perc_green_tracts + area_total_tracts + popu_dens_tracts + white_pct_acs_10 x med_hh_inc_acs_10 + med_hh_inc_acs_10^2 + perc_green_tracts^2 + area_total_tracts^2 + popu_dens_tracts^2 | 12 | 2713.58 | 2.68 | 0.04 | -1344.57 | 0.8 |
| BOS_67 | perc_green_tracts + area_total_tracts x popu_dens_tracts + white_pct_acs_10 + med_hh_inc_acs_10 | 8 | 2713.91 | 3.02 | 0.03 | -1348.86 | 0.83 |
| BOS_190 | perc_green_tracts + area_total_tracts x popu_dens_tracts + white_pct_acs_10 + med_hh_inc_acs_10 + white_pct_acs_10^2 + med_hh_inc_acs_10^2 + perc_green_tracts^2 + area_total_tracts^2 + popu_dens_tracts^2 | 13 | 2714.82 | 3.93 | 0.02 | -1344.16 | 0.85 |
| BOS_179 | perc_green_tracts + area_total_tracts + popu_dens_tracts + white_pct_acs_10 + med_hh_inc_acs_10 + med_hh_inc_acs_10^2 + perc_green_tracts^2 + area_total_tracts^2 + popu_dens_tracts^2 | 11 | 2715.13 | 4.23 | 0.02 | -1346.38 | 0.87 |
| BOS_169 | perc_green_tracts + area_total_tracts + popu_dens_tracts + white_pct_acs_10 + med_hh_inc_acs_10 + white_pct_acs_10^2 + perc_green_tracts^2 + area_total_tracts^2 + popu_dens_tracts^2 | 11 | 2715.16 | 4.26 | 0.02 | -1346.4 | 0.89 |
| BOS_103 | perc_green_tracts + area_total_tracts x popu_dens_tracts + white_pct_acs_10 x med_hh_inc_acs_10 + white_pct_acs_10^2 | 10 | 2715.27 | 4.37 | 0.02 | -1347.48 | 0.91 |
| BOS_85 | perc_green_tracts + area_total_tracts x popu_dens_tracts + white_pct_acs_10 + med_hh_inc_acs_10 + med_hh_inc_acs_10^2 | 9 | 2715.69 | 4.8 | 0.01 | -1348.72 | 0.92 |
| BOS_76 | perc_green_tracts + area_total_tracts x popu_dens_tracts + white_pct_acs_10 + med_hh_inc_acs_10 + white_pct_acs_10^2 | 9 | 2715.75 | 4.85 | 0.01 | -1348.75 | 0.93 |
| BOS_121 | perc_green_tracts + area_total_tracts x popu_dens_tracts + white_pct_acs_10 x med_hh_inc_acs_10 + white_pct_acs_10^2 + med_hh_inc_acs_10^2 | 11 | 2715.93 | 5.04 | 0.01 | -1346.79 | 0.94 |
| BOS_112 | perc_green_tracts + area_total_tracts x popu_dens_tracts + white_pct_acs_10 x med_hh_inc_acs_10 + med_hh_inc_acs_10^2 | 10 | 2716.12 | 5.22 | 0.01 | -1347.91 | 0.96 |
| BOS_66 | perc_green_tracts + area_total_tracts + popu_dens_tracts + white_pct_acs_10 + med_hh_inc_acs_10 | 7 | 2716.92 | 6.02 | 0.01 | -1351.38 | 0.96 |
| BOS_189 | perc_green_tracts + area_total_tracts + popu_dens_tracts + white_pct_acs_10 + med_hh_inc_acs_10 + white_pct_acs_10^2 + med_hh_inc_acs_10^2 + perc_green_tracts^2 + area_total_tracts^2 + popu_dens_tracts^2 | 12 | 2717.16 | 6.26 | 0.01 | -1346.37 | 0.97 |
| BOS_94 | perc_green_tracts + area_total_tracts x popu_dens_tracts + white_pct_acs_10 + med_hh_inc_acs_10 + white_pct_acs_10^2 + med_hh_inc_acs_10^2 | 10 | 2717.54 | 6.64 | 0.01 | -1348.62 | 0.97 |
| BOS_102 | perc_green_tracts + area_total_tracts + popu_dens_tracts + white_pct_acs_10 x med_hh_inc_acs_10 + white_pct_acs_10^2 | 9 | 2718.09 | 7.19 | 0 | -1349.92 | 0.98 |
| BOS_75 | perc_green_tracts + area_total_tracts + popu_dens_tracts + white_pct_acs_10 + med_hh_inc_acs_10 + white_pct_acs_10^2 | 8 | 2718.86 | 7.97 | 0 | -1351.33 | 0.98 |
| BOS_84 | perc_green_tracts + area_total_tracts + popu_dens_tracts + white_pct_acs_10 + med_hh_inc_acs_10 + med_hh_inc_acs_10^2 | 8 | 2718.92 | 8.03 | 0 | -1351.36 | 0.98 |
| BOS_111 | perc_green_tracts + area_total_tracts + popu_dens_tracts + white_pct_acs_10 x med_hh_inc_acs_10 + med_hh_inc_acs_10^2 | 9 | 2719.17 | 8.27 | 0 | -1350.46 | 0.99 |
| BOS_120 | perc_green_tracts + area_total_tracts + popu_dens_tracts + white_pct_acs_10 x med_hh_inc_acs_10 + white_pct_acs_10^2 + med_hh_inc_acs_10^2 | 10 | 2719.38 | 8.49 | 0 | -1349.54 | 0.99 |
| BOS_130 | perc_green_tracts + area_total_tracts x popu_dens_tracts + white_pct_acs_10 + perc_green_tracts^2 + area_total_tracts^2 + popu_dens_tracts^2 | 10 | 2719.59 | 8.69 | 0 | -1349.64 | 0.99 |
| BOS_194 | perc_green_tracts + area_total_tracts + white_pct_acs_10 x med_hh_inc_acs_10 + white_pct_acs_10^2 + perc_green_tracts^2 + area_total_tracts^2 | 10 | 2720.6 | 9.7 | 0 | -1350.15 | 0.99 |
| BOS_93 | perc_green_tracts + area_total_tracts + popu_dens_tracts + white_pct_acs_10 + med_hh_inc_acs_10 + white_pct_acs_10^2 + med_hh_inc_acs_10^2 | 9 | 2720.88 | 9.98 | 0 | -1351.32 | 0.99 |
| BOS_129 | perc_green_tracts + area_total_tracts + popu_dens_tracts + white_pct_acs_10 + perc_green_tracts^2 + area_total_tracts^2 + popu_dens_tracts^2 | 9 | 2721.18 | 10.28 | 0 | -1351.47 | 0.99 |
| BOS_154 | perc_green_tracts + area_total_tracts + white_pct_acs_10 + med_hh_inc_acs_10 + perc_green_tracts^2 + area_total_tracts^2 | 8 | 2721.37 | 10.48 | 0 | -1352.59 | 0.99 |
| BOS_140 | perc_green_tracts + area_total_tracts x popu_dens_tracts + white_pct_acs_10 + white_pct_acs_10^2 + perc_green_tracts^2 + area_total_tracts^2 + popu_dens_tracts^2 | 11 | 2721.58 | 10.69 | 0 | -1349.61 | 0.99 |
| BOS_214 | perc_green_tracts + area_total_tracts + white_pct_acs_10 x med_hh_inc_acs_10 + white_pct_acs_10^2 + med_hh_inc_acs_10^2 + perc_green_tracts^2 + area_total_tracts^2 | 11 | 2721.6 | 10.71 | 0 | -1349.62 | 0.99 |
| BOS_204 | perc_green_tracts + area_total_tracts + white_pct_acs_10 x med_hh_inc_acs_10 + med_hh_inc_acs_10^2 + perc_green_tracts^2 + area_total_tracts^2 | 10 | 2722.05 | 11.15 | 0 | -1350.88 | 1 |
| BOS_40 | perc_green_tracts + area_total_tracts x popu_dens_tracts + white_pct_acs_10 | 7 | 2722.55 | 11.65 | 0 | -1354.2 | 1 |
| BOS_195 | perc_green_tracts x area_total_tracts + white_pct_acs_10 x med_hh_inc_acs_10 + white_pct_acs_10^2 + perc_green_tracts^2 + area_total_tracts^2 | 11 | 2722.63 | 11.73 | 0 | -1350.13 | 1 |
| BOS_139 | perc_green_tracts + area_total_tracts + popu_dens_tracts + white_pct_acs_10 + white_pct_acs_10^2 + perc_green_tracts^2 + area_total_tracts^2 + popu_dens_tracts^2 | 10 | 2723.23 | 12.33 | 0 | -1351.47 | 1 |
| BOS_150 | perc_green_tracts + area_total_tracts x popu_dens_tracts + med_hh_inc_acs_10 + med_hh_inc_acs_10^2 + perc_green_tracts^2 + area_total_tracts^2 + popu_dens_tracts^2 | 11 | 2723.3 | 12.41 | 0 | -1350.47 | 1 |
| BOS_164 | perc_green_tracts + area_total_tracts + white_pct_acs_10 + med_hh_inc_acs_10 + white_pct_acs_10^2 + perc_green_tracts^2 + area_total_tracts^2 | 9 | 2723.35 | 12.46 | 0 | -1352.55 | 1 |
| BOS_174 | perc_green_tracts + area_total_tracts + white_pct_acs_10 + med_hh_inc_acs_10 + med_hh_inc_acs_10^2 + perc_green_tracts^2 + area_total_tracts^2 | 9 | 2723.41 | 12.51 | 0 | -1352.58 | 1 |
| BOS_155 | perc_green_tracts x area_total_tracts + white_pct_acs_10 + med_hh_inc_acs_10 + perc_green_tracts^2 + area_total_tracts^2 | 9 | 2723.42 | 12.52 | 0 | -1352.59 | 1 |
| BOS_215 | perc_green_tracts x area_total_tracts + white_pct_acs_10 x med_hh_inc_acs_10 + white_pct_acs_10^2 + med_hh_inc_acs_10^2 + perc_green_tracts^2 + area_total_tracts^2 | 12 | 2723.6 | 12.7 | 0 | -1349.59 | 1 |
| BOS_205 | perc_green_tracts x area_total_tracts + white_pct_acs_10 x med_hh_inc_acs_10 + med_hh_inc_acs_10^2 + perc_green_tracts^2 + area_total_tracts^2 | 11 | 2724.1 | 13.2 | 0 | -1350.87 | 1 |
| BOS_49 | perc_green_tracts + area_total_tracts x popu_dens_tracts + white_pct_acs_10 + white_pct_acs_10^2 | 8 | 2724.4 | 13.51 | 0 | -1354.1 | 1 |
| BOS_149 | perc_green_tracts + area_total_tracts + popu_dens_tracts + med_hh_inc_acs_10 + med_hh_inc_acs_10^2 + perc_green_tracts^2 + area_total_tracts^2 + popu_dens_tracts^2 | 10 | 2724.67 | 13.77 | 0 | -1352.18 | 1 |
| BOS_58 | perc_green_tracts + area_total_tracts x popu_dens_tracts + med_hh_inc_acs_10 + med_hh_inc_acs_10^2 | 8 | 2724.87 | 13.98 | 0 | -1354.34 | 1 |
| BOS_97 | perc_green_tracts + area_total_tracts + white_pct_acs_10 x med_hh_inc_acs_10 + white_pct_acs_10^2 | 8 | 2724.92 | 14.03 | 0 | -1354.36 | 1 |
| BOS_61 | perc_green_tracts + area_total_tracts + white_pct_acs_10 + med_hh_inc_acs_10 | 6 | 2725.19 | 14.29 | 0 | -1356.54 | 1 |
| BOS_62 | perc_green_tracts x area_total_tracts + white_pct_acs_10 + med_hh_inc_acs_10 | 7 | 2725.2 | 14.3 | 0 | -1355.52 | 1 |
| BOS_98 | perc_green_tracts x area_total_tracts + white_pct_acs_10 x med_hh_inc_acs_10 + white_pct_acs_10^2 | 9 | 2725.36 | 14.46 | 0 | -1353.56 | 1 |
| BOS_184 | perc_green_tracts + area_total_tracts + white_pct_acs_10 + med_hh_inc_acs_10 + white_pct_acs_10^2 + med_hh_inc_acs_10^2 + perc_green_tracts^2 + area_total_tracts^2 | 10 | 2725.4 | 14.5 | 0 | -1352.55 | 1 |
| BOS_165 | perc_green_tracts x area_total_tracts + white_pct_acs_10 + med_hh_inc_acs_10 + white_pct_acs_10^2 + perc_green_tracts^2 + area_total_tracts^2 | 10 | 2725.41 | 14.51 | 0 | -1352.55 | 1 |
| BOS_175 | perc_green_tracts x area_total_tracts + white_pct_acs_10 + med_hh_inc_acs_10 + med_hh_inc_acs_10^2 + perc_green_tracts^2 + area_total_tracts^2 | 10 | 2725.46 | 14.57 | 0 | -1352.58 | 1 |
| BOS_39 | perc_green_tracts + area_total_tracts + popu_dens_tracts + white_pct_acs_10 | 6 | 2725.49 | 14.59 | 0 | -1356.69 | 1 |
| BOS_115 | perc_green_tracts + area_total_tracts + white_pct_acs_10 x med_hh_inc_acs_10 + white_pct_acs_10^2 + med_hh_inc_acs_10^2 | 9 | 2726.58 | 15.69 | 0 | -1354.17 | 1 |
| BOS_70 | perc_green_tracts + area_total_tracts + white_pct_acs_10 + med_hh_inc_acs_10 + white_pct_acs_10^2 | 7 | 2726.67 | 15.77 | 0 | -1356.26 | 1 |
| BOS_71 | perc_green_tracts x area_total_tracts + white_pct_acs_10 + med_hh_inc_acs_10 + white_pct_acs_10^2 | 8 | 2726.91 | 16.01 | 0 | -1355.35 | 1 |
| BOS_116 | perc_green_tracts x area_total_tracts + white_pct_acs_10 x med_hh_inc_acs_10 + white_pct_acs_10^2 + med_hh_inc_acs_10^2 | 10 | 2727.01 | 16.11 | 0 | -1353.35 | 1 |
| BOS_79 | perc_green_tracts + area_total_tracts + white_pct_acs_10 + med_hh_inc_acs_10 + med_hh_inc_acs_10^2 | 7 | 2727.21 | 16.31 | 0 | -1356.53 | 1 |
| BOS_80 | perc_green_tracts x area_total_tracts + white_pct_acs_10 + med_hh_inc_acs_10 + med_hh_inc_acs_10^2 | 8 | 2727.23 | 16.33 | 0 | -1355.51 | 1 |
| BOS_48 | perc_green_tracts + area_total_tracts + popu_dens_tracts + white_pct_acs_10 + white_pct_acs_10^2 | 7 | 2727.45 | 16.55 | 0 | -1356.65 | 1 |
| BOS_185 | perc_green_tracts x area_total_tracts + white_pct_acs_10 + med_hh_inc_acs_10 + white_pct_acs_10^2 + med_hh_inc_acs_10^2 + perc_green_tracts^2 + area_total_tracts^2 | 11 | 2727.46 | 16.56 | 0 | -1352.55 | 1 |
| BOS_57 | perc_green_tracts + area_total_tracts + popu_dens_tracts + med_hh_inc_acs_10 + med_hh_inc_acs_10^2 | 7 | 2727.5 | 16.6 | 0 | -1356.67 | 1 |
| BOS_107 | perc_green_tracts x area_total_tracts + white_pct_acs_10 x med_hh_inc_acs_10 + med_hh_inc_acs_10^2 | 9 | 2727.64 | 16.75 | 0 | -1354.7 | 1 |
| BOS_106 | perc_green_tracts + area_total_tracts + white_pct_acs_10 x med_hh_inc_acs_10 + med_hh_inc_acs_10^2 | 8 | 2727.78 | 16.88 | 0 | -1355.79 | 1 |
| BOS_88 | perc_green_tracts + area_total_tracts + white_pct_acs_10 + med_hh_inc_acs_10 + white_pct_acs_10^2 + med_hh_inc_acs_10^2 | 8 | 2728.69 | 17.8 | 0 | -1356.25 | 1 |
| BOS_89 | perc_green_tracts x area_total_tracts + white_pct_acs_10 + med_hh_inc_acs_10 + white_pct_acs_10^2 + med_hh_inc_acs_10^2 | 9 | 2728.94 | 18.04 | 0 | -1355.35 | 1 |
| BOS_158 | perc_green_tracts x popu_dens_tracts + white_pct_acs_10 + med_hh_inc_acs_10 + perc_green_tracts^2 + popu_dens_tracts^2 | 9 | 2728.95 | 18.05 | 0 | -1355.35 | 1 |
| BOS_124 | perc_green_tracts + area_total_tracts + white_pct_acs_10 + perc_green_tracts^2 + area_total_tracts^2 | 7 | 2729.83 | 18.93 | 0 | -1357.84 | 1 |
| BOS_198 | perc_green_tracts x popu_dens_tracts + white_pct_acs_10 x med_hh_inc_acs_10 + white_pct_acs_10^2 + perc_green_tracts^2 + popu_dens_tracts^2 | 11 | 2729.97 | 19.08 | 0 | -1353.81 | 1 |
| BOS_168 | perc_green_tracts x popu_dens_tracts + white_pct_acs_10 + med_hh_inc_acs_10 + white_pct_acs_10^2 + perc_green_tracts^2 + popu_dens_tracts^2 | 10 | 2730.23 | 19.33 | 0 | -1354.96 | 1 |
| BOS_178 | perc_green_tracts x popu_dens_tracts + white_pct_acs_10 + med_hh_inc_acs_10 + med_hh_inc_acs_10^2 + perc_green_tracts^2 + popu_dens_tracts^2 | 10 | 2731 | 20.1 | 0 | -1355.35 | 1 |
| BOS_218 | perc_green_tracts x popu_dens_tracts + white_pct_acs_10 x med_hh_inc_acs_10 + white_pct_acs_10^2 + med_hh_inc_acs_10^2 + perc_green_tracts^2 + popu_dens_tracts^2 | 12 | 2731.57 | 20.68 | 0 | -1353.57 | 1 |
| BOS_125 | perc_green_tracts x area_total_tracts + white_pct_acs_10 + perc_green_tracts^2 + area_total_tracts^2 | 8 | 2731.84 | 20.94 | 0 | -1357.82 | 1 |
| BOS_134 | perc_green_tracts + area_total_tracts + white_pct_acs_10 + white_pct_acs_10^2 + perc_green_tracts^2 + area_total_tracts^2 | 8 | 2731.84 | 20.94 | 0 | -1357.82 | 1 |
| BOS_157 | perc_green_tracts + popu_dens_tracts + white_pct_acs_10 + med_hh_inc_acs_10 + perc_green_tracts^2 + popu_dens_tracts^2 | 8 | 2731.9 | 21 | 0 | -1357.85 | 1 |
| BOS_144 | perc_green_tracts + area_total_tracts + med_hh_inc_acs_10 + med_hh_inc_acs_10^2 + perc_green_tracts^2 + area_total_tracts^2 | 8 | 2732.15 | 21.25 | 0 | -1357.98 | 1 |
| BOS_188 | perc_green_tracts x popu_dens_tracts + white_pct_acs_10 + med_hh_inc_acs_10 + white_pct_acs_10^2 + med_hh_inc_acs_10^2 + perc_green_tracts^2 + popu_dens_tracts^2 | 11 | 2732.28 | 21.38 | 0 | -1354.96 | 1 |
| BOS_208 | perc_green_tracts x popu_dens_tracts + white_pct_acs_10 x med_hh_inc_acs_10 + med_hh_inc_acs_10^2 + perc_green_tracts^2 + popu_dens_tracts^2 | 11 | 2732.4 | 21.5 | 0 | -1355.02 | 1 |
| BOS_167 | perc_green_tracts + popu_dens_tracts + white_pct_acs_10 + med_hh_inc_acs_10 + white_pct_acs_10^2 + perc_green_tracts^2 + popu_dens_tracts^2 | 9 | 2732.62 | 21.72 | 0 | -1357.19 | 1 |
| BOS_197 | perc_green_tracts + popu_dens_tracts + white_pct_acs_10 x med_hh_inc_acs_10 + white_pct_acs_10^2 + perc_green_tracts^2 + popu_dens_tracts^2 | 10 | 2732.82 | 21.92 | 0 | -1356.26 | 1 |
| BOS_64 | perc_green_tracts + popu_dens_tracts + white_pct_acs_10 + med_hh_inc_acs_10 | 6 | 2733.63 | 22.73 | 0 | -1360.76 | 1 |
| BOS_135 | perc_green_tracts x area_total_tracts + white_pct_acs_10 + white_pct_acs_10^2 + perc_green_tracts^2 + area_total_tracts^2 | 9 | 2733.86 | 22.96 | 0 | -1357.81 | 1 |
| BOS_65 | perc_green_tracts x popu_dens_tracts + white_pct_acs_10 + med_hh_inc_acs_10 | 7 | 2733.94 | 23.04 | 0 | -1359.89 | 1 |
| BOS_177 | perc_green_tracts + popu_dens_tracts + white_pct_acs_10 + med_hh_inc_acs_10 + med_hh_inc_acs_10^2 + perc_green_tracts^2 + popu_dens_tracts^2 | 9 | 2733.95 | 23.05 | 0 | -1357.85 | 1 |
| BOS_145 | perc_green_tracts x area_total_tracts + med_hh_inc_acs_10 + med_hh_inc_acs_10^2 + perc_green_tracts^2 + area_total_tracts^2 | 9 | 2734.08 | 23.18 | 0 | -1357.92 | 1 |
| BOS_73 | perc_green_tracts + popu_dens_tracts + white_pct_acs_10 + med_hh_inc_acs_10 + white_pct_acs_10^2 | 7 | 2734.27 | 23.37 | 0 | -1360.06 | 1 |
| BOS_161 | area_total_tracts x popu_dens_tracts + white_pct_acs_10 + med_hh_inc_acs_10 + perc_green_tracts^2 + area_total_tracts^2 + popu_dens_tracts^2 | 10 | 2734.47 | 23.57 | 0 | -1357.08 | 1 |
| BOS_217 | perc_green_tracts + popu_dens_tracts + white_pct_acs_10 x med_hh_inc_acs_10 + white_pct_acs_10^2 + med_hh_inc_acs_10^2 + perc_green_tracts^2 + popu_dens_tracts^2 | 11 | 2734.58 | 23.69 | 0 | -1356.11 | 1 |
| BOS_187 | perc_green_tracts + popu_dens_tracts + white_pct_acs_10 + med_hh_inc_acs_10 + white_pct_acs_10^2 + med_hh_inc_acs_10^2 + perc_green_tracts^2 + popu_dens_tracts^2 | 10 | 2734.67 | 23.78 | 0 | -1357.19 | 1 |
| BOS_74 | perc_green_tracts x popu_dens_tracts + white_pct_acs_10 + med_hh_inc_acs_10 + white_pct_acs_10^2 | 8 | 2734.79 | 23.89 | 0 | -1359.3 | 1 |
| BOS_100 | perc_green_tracts + popu_dens_tracts + white_pct_acs_10 x med_hh_inc_acs_10 + white_pct_acs_10^2 | 8 | 2735.01 | 24.11 | 0 | -1359.41 | 1 |
| BOS_201 | area_total_tracts x popu_dens_tracts + white_pct_acs_10 x med_hh_inc_acs_10 + white_pct_acs_10^2 + perc_green_tracts^2 + area_total_tracts^2 + popu_dens_tracts^2 | 12 | 2735.05 | 24.16 | 0 | -1355.31 | 1 |
| BOS_101 | perc_green_tracts x popu_dens_tracts + white_pct_acs_10 x med_hh_inc_acs_10 + white_pct_acs_10^2 | 9 | 2735.38 | 24.48 | 0 | -1358.57 | 1 |
| BOS_82 | perc_green_tracts + popu_dens_tracts + white_pct_acs_10 + med_hh_inc_acs_10 + med_hh_inc_acs_10^2 | 7 | 2735.67 | 24.77 | 0 | -1360.76 | 1 |
| BOS_207 | perc_green_tracts + popu_dens_tracts + white_pct_acs_10 x med_hh_inc_acs_10 + med_hh_inc_acs_10^2 + perc_green_tracts^2 + popu_dens_tracts^2 | 10 | 2735.78 | 24.88 | 0 | -1357.74 | 1 |
| BOS_2 | white_pct_acs_10 x med_hh_inc_acs_10 + white_pct_acs_10^2 + med_hh_inc_acs_10^2 + area_total_tracts x popu_dens_tracts + perc_green_tracts^2 + area_total_tracts^2 + popu_dens_tracts^2 | 13 | 2735.81 | 24.91 | 0 | -1354.65 | 1 |
| BOS_83 | perc_green_tracts x popu_dens_tracts + white_pct_acs_10 + med_hh_inc_acs_10 + med_hh_inc_acs_10^2 | 8 | 2735.98 | 25.09 | 0 | -1359.89 | 1 |
| BOS_171 | area_total_tracts x popu_dens_tracts + white_pct_acs_10 + med_hh_inc_acs_10 + white_pct_acs_10^2 + perc_green_tracts^2 + area_total_tracts^2 + popu_dens_tracts^2 | 11 | 2736.22 | 25.32 | 0 | -1356.93 | 1 |
| BOS_91 | perc_green_tracts + popu_dens_tracts + white_pct_acs_10 + med_hh_inc_acs_10 + white_pct_acs_10^2 + med_hh_inc_acs_10^2 | 8 | 2736.32 | 25.42 | 0 | -1360.06 | 1 |
| BOS_128 | perc_green_tracts x popu_dens_tracts + white_pct_acs_10 + perc_green_tracts^2 + popu_dens_tracts^2 | 8 | 2736.33 | 25.43 | 0 | -1360.07 | 1 |
| BOS_181 | area_total_tracts x popu_dens_tracts + white_pct_acs_10 + med_hh_inc_acs_10 + med_hh_inc_acs_10^2 + perc_green_tracts^2 + area_total_tracts^2 + popu_dens_tracts^2 | 11 | 2736.36 | 25.47 | 0 | -1357 | 1 |
| BOS_211 | area_total_tracts x popu_dens_tracts + white_pct_acs_10 x med_hh_inc_acs_10 + med_hh_inc_acs_10^2 + perc_green_tracts^2 + area_total_tracts^2 + popu_dens_tracts^2 | 12 | 2736.47 | 25.57 | 0 | -1356.02 | 1 |
| BOS_35 | perc_green_tracts x area_total_tracts + white_pct_acs_10 | 6 | 2736.62 | 25.72 | 0 | -1362.25 | 1 |
| BOS_92 | perc_green_tracts x popu_dens_tracts + white_pct_acs_10 + med_hh_inc_acs_10 + white_pct_acs_10^2 + med_hh_inc_acs_10^2 | 9 | 2736.84 | 25.94 | 0 | -1359.29 | 1 |
| BOS_118 | perc_green_tracts + popu_dens_tracts + white_pct_acs_10 x med_hh_inc_acs_10 + white_pct_acs_10^2 + med_hh_inc_acs_10^2 | 9 | 2736.9 | 26 | 0 | -1359.33 | 1 |
| BOS_34 | perc_green_tracts + area_total_tracts + white_pct_acs_10 | 5 | 2736.99 | 26.1 | 0 | -1363.46 | 1 |
| BOS_53 | perc_green_tracts x area_total_tracts + med_hh_inc_acs_10 + med_hh_inc_acs_10^2 | 7 | 2737.2 | 26.3 | 0 | -1361.52 | 1 |
| BOS_119 | perc_green_tracts x popu_dens_tracts + white_pct_acs_10 x med_hh_inc_acs_10 + white_pct_acs_10^2 + med_hh_inc_acs_10^2 | 10 | 2737.26 | 26.36 | 0 | -1358.48 | 1 |
| BOS_109 | perc_green_tracts + popu_dens_tracts + white_pct_acs_10 x med_hh_inc_acs_10 + med_hh_inc_acs_10^2 | 8 | 2737.65 | 26.75 | 0 | -1360.73 | 1 |
| BOS_138 | perc_green_tracts x popu_dens_tracts + white_pct_acs_10 + white_pct_acs_10^2 + perc_green_tracts^2 + popu_dens_tracts^2 | 9 | 2737.82 | 26.92 | 0 | -1359.79 | 1 |
| BOS_110 | perc_green_tracts x popu_dens_tracts + white_pct_acs_10 x med_hh_inc_acs_10 + med_hh_inc_acs_10^2 | 9 | 2737.91 | 27.01 | 0 | -1359.83 | 1 |
| BOS_191 | area_total_tracts x popu_dens_tracts + white_pct_acs_10 + med_hh_inc_acs_10 + white_pct_acs_10^2 + med_hh_inc_acs_10^2 + perc_green_tracts^2 + area_total_tracts^2 + popu_dens_tracts^2 | 12 | 2738.12 | 27.23 | 0 | -1356.85 | 1 |
| BOS_44 | perc_green_tracts x area_total_tracts + white_pct_acs_10 + white_pct_acs_10^2 | 7 | 2738.3 | 27.4 | 0 | -1362.07 | 1 |
| BOS_43 | perc_green_tracts + area_total_tracts + white_pct_acs_10 + white_pct_acs_10^2 | 6 | 2738.42 | 27.52 | 0 | -1363.15 | 1 |
| BOS_20 | perc_green_tracts + area_total_tracts x popu_dens_tracts + perc_green_tracts^2 + area_total_tracts^2 + popu_dens_tracts^2 | 9 | 2739.06 | 28.16 | 0 | -1360.4 | 1 |
| BOS_52 | perc_green_tracts + area_total_tracts + med_hh_inc_acs_10 + med_hh_inc_acs_10^2 | 6 | 2739.23 | 28.33 | 0 | -1363.56 | 1 |
| BOS_19 | perc_green_tracts + area_total_tracts + popu_dens_tracts + perc_green_tracts^2 + area_total_tracts^2 + popu_dens_tracts^2 | 8 | 2740.23 | 29.33 | 0 | -1362.02 | 1 |
| BOS_192 | perc_green_tracts + white_pct_acs_10 x med_hh_inc_acs_10 + white_pct_acs_10^2 + perc_green_tracts^2 | 8 | 2742.27 | 31.38 | 0 | -1363.04 | 1 |
| BOS_127 | perc_green_tracts + popu_dens_tracts + white_pct_acs_10 + perc_green_tracts^2 + popu_dens_tracts^2 | 7 | 2742.6 | 31.7 | 0 | -1364.22 | 1 |
| BOS_162 | perc_green_tracts + white_pct_acs_10 + med_hh_inc_acs_10 + white_pct_acs_10^2 + perc_green_tracts^2 | 7 | 2742.62 | 31.72 | 0 | -1364.23 | 1 |
| BOS_148 | perc_green_tracts x popu_dens_tracts + med_hh_inc_acs_10 + med_hh_inc_acs_10^2 + perc_green_tracts^2 + popu_dens_tracts^2 | 9 | 2742.92 | 32.02 | 0 | -1362.34 | 1 |
| BOS_152 | perc_green_tracts + white_pct_acs_10 + med_hh_inc_acs_10 + perc_green_tracts^2 | 6 | 2743.12 | 32.23 | 0 | -1365.5 | 1 |
| BOS_137 | perc_green_tracts + popu_dens_tracts + white_pct_acs_10 + white_pct_acs_10^2 + perc_green_tracts^2 + popu_dens_tracts^2 | 8 | 2743.46 | 32.56 | 0 | -1363.63 | 1 |
| BOS_38 | perc_green_tracts x popu_dens_tracts + white_pct_acs_10 | 6 | 2743.66 | 32.76 | 0 | -1365.77 | 1 |
| BOS_11 | perc_green_tracts + area_total_tracts x popu_dens_tracts | 6 | 2743.98 | 33.08 | 0 | -1365.93 | 1 |
| BOS_212 | perc_green_tracts + white_pct_acs_10 x med_hh_inc_acs_10 + white_pct_acs_10^2 + med_hh_inc_acs_10^2 + perc_green_tracts^2 | 9 | 2744.24 | 33.35 | 0 | -1363 | 1 |
| BOS_47 | perc_green_tracts x popu_dens_tracts + white_pct_acs_10 + white_pct_acs_10^2 | 7 | 2744.51 | 33.61 | 0 | -1365.18 | 1 |
| BOS_182 | perc_green_tracts + white_pct_acs_10 + med_hh_inc_acs_10 + white_pct_acs_10^2 + med_hh_inc_acs_10^2 + perc_green_tracts^2 | 8 | 2744.57 | 33.68 | 0 | -1364.19 | 1 |
| BOS_37 | perc_green_tracts + popu_dens_tracts + white_pct_acs_10 | 5 | 2744.94 | 34.04 | 0 | -1367.43 | 1 |
| BOS_172 | perc_green_tracts + white_pct_acs_10 + med_hh_inc_acs_10 + med_hh_inc_acs_10^2 + perc_green_tracts^2 | 7 | 2745.06 | 34.16 | 0 | -1365.45 | 1 |
| BOS_196 | area_total_tracts + white_pct_acs_10 x med_hh_inc_acs_10 + white_pct_acs_10^2 + perc_green_tracts^2 + area_total_tracts^2 | 9 | 2745.35 | 34.45 | 0 | -1363.55 | 1 |
| BOS_46 | perc_green_tracts + popu_dens_tracts + white_pct_acs_10 + white_pct_acs_10^2 | 6 | 2745.49 | 34.6 | 0 | -1366.69 | 1 |
| BOS_131 | area_total_tracts x popu_dens_tracts + white_pct_acs_10 + perc_green_tracts^2 + area_total_tracts^2 + popu_dens_tracts^2 | 8 | 2745.66 | 34.76 | 0 | -1363.71 | 1 |
| BOS_147 | perc_green_tracts + popu_dens_tracts + med_hh_inc_acs_10 + med_hh_inc_acs_10^2 + perc_green_tracts^2 + popu_dens_tracts^2 | 9 | 2745.66 | 34.76 | 0 | -1364.73 | 1 |
| BOS_156 | area_total_tracts + white_pct_acs_10 + med_hh_inc_acs_10 + perc_green_tracts^2 + area_total_tracts^2 | 7 | 2746 | 35.11 | 0 | -1365.92 | 1 |
| BOS_216 | area_total_tracts + white_pct_acs_10 x med_hh_inc_acs_10 + white_pct_acs_10^2 + med_hh_inc_acs_10^2 + perc_green_tracts^2 + area_total_tracts^2 | 10 | 2746.86 | 35.96 | 0 | -1363.28 | 1 |
| BOS_69 | perc_green_tracts + white_pct_acs_10 + med_hh_inc_acs_10 + white_pct_acs_10^2 | 6 | 2746.95 | 36.05 | 0 | -1367.42 | 1 |
| BOS_96 | perc_green_tracts + white_pct_acs_10 x med_hh_inc_acs_10 + white_pct_acs_10^2 | 7 | 2747.03 | 36.13 | 0 | -1366.44 | 1 |
| BOS_202 | perc_green_tracts + white_pct_acs_10 x med_hh_inc_acs_10 + med_hh_inc_acs_10^2 + perc_green_tracts^2 | 8 | 2747.03 | 36.13 | 0 | -1365.42 | 1 |
| BOS_10 | perc_green_tracts + area_total_tracts + popu_dens_tracts | 5 | 2747.29 | 36.39 | 0 | -1368.6 | 1 |
| BOS_141 | area_total_tracts x popu_dens_tracts + white_pct_acs_10 + white_pct_acs_10^2 + perc_green_tracts^2 + area_total_tracts^2 + popu_dens_tracts^2 | 10 | 2747.56 | 36.67 | 0 | -1363.63 | 1 |
| BOS_206 | area_total_tracts + white_pct_acs_10 x med_hh_inc_acs_10 + med_hh_inc_acs_10^2 + perc_green_tracts^2 + area_total_tracts^2 | 9 | 2747.72 | 36.83 | 0 | -1364.74 | 1 |
| BOS_166 | area_total_tracts + white_pct_acs_10 + med_hh_inc_acs_10 + white_pct_acs_10^2 + perc_green_tracts^2 + area_total_tracts^2 | 8 | 2747.76 | 36.86 | 0 | -1365.78 | 1 |
| BOS_151 | area_total_tracts x popu_dens_tracts + med_hh_inc_acs_10 + med_hh_inc_acs_10^2 + perc_green_tracts^2 + area_total_tracts^2 + popu_dens_tracts^2 | 10 | 2747.77 | 36.87 | 0 | -1363.73 | 1 |
| BOS_55 | perc_green_tracts + popu_dens_tracts + med_hh_inc_acs_10 + med_hh_inc_acs_10^2 | 6 | 2747.96 | 37.06 | 0 | -1367.92 | 1 |
| BOS_176 | area_total_tracts + white_pct_acs_10 + med_hh_inc_acs_10 + med_hh_inc_acs_10^2 + perc_green_tracts^2 + area_total_tracts^2 | 8 | 2748.03 | 37.13 | 0 | -1365.92 | 1 |
| BOS_60 | perc_green_tracts + white_pct_acs_10 + med_hh_inc_acs_10 | 5 | 2748.43 | 37.53 | 0 | -1369.18 | 1 |
| BOS_87 | perc_green_tracts + white_pct_acs_10 + med_hh_inc_acs_10 + white_pct_acs_10^2 + med_hh_inc_acs_10^2 | 7 | 2748.75 | 37.85 | 0 | -1367.3 | 1 |
| BOS_56 | perc_green_tracts x popu_dens_tracts + med_hh_inc_acs_10 + med_hh_inc_acs_10^2 | 7 | 2748.88 | 37.98 | 0 | -1367.36 | 1 |
| BOS_114 | perc_green_tracts + white_pct_acs_10 x med_hh_inc_acs_10 + white_pct_acs_10^2 + med_hh_inc_acs_10^2 | 8 | 2749.07 | 38.17 | 0 | -1366.44 | 1 |
| BOS_186 | area_total_tracts + white_pct_acs_10 + med_hh_inc_acs_10 + white_pct_acs_10^2 + med_hh_inc_acs_10^2 + perc_green_tracts^2 + area_total_tracts^2 | 9 | 2749.79 | 38.9 | 0 | -1365.77 | 1 |
| BOS_78 | perc_green_tracts + white_pct_acs_10 + med_hh_inc_acs_10 + med_hh_inc_acs_10^2 | 6 | 2750.19 | 39.29 | 0 | -1369.04 | 1 |
| BOS_14 | perc_green_tracts + area_total_tracts + perc_green_tracts^2 + area_total_tracts^2 | 6 | 2751.42 | 40.53 | 0 | -1369.65 | 1 |
| BOS_105 | perc_green_tracts + white_pct_acs_10 x med_hh_inc_acs_10 + med_hh_inc_acs_10^2 | 7 | 2752.22 | 41.32 | 0 | -1369.03 | 1 |
| BOS_15 | perc_green_tracts x area_total_tracts + perc_green_tracts^2 + area_total_tracts^2 | 7 | 2753.44 | 42.54 | 0 | -1369.64 | 1 |
| BOS_132 | perc_green_tracts + white_pct_acs_10 + white_pct_acs_10^2 + perc_green_tracts^2 | 6 | 2756.63 | 45.73 | 0 | -1372.26 | 1 |
| BOS_122 | perc_green_tracts + white_pct_acs_10 + perc_green_tracts^2 | 5 | 2757.48 | 46.58 | 0 | -1373.7 | 1 |
| BOS_126 | area_total_tracts + white_pct_acs_10 + perc_green_tracts^2 + area_total_tracts^2 | 6 | 2758.52 | 47.62 | 0 | -1373.2 | 1 |
| BOS_146 | area_total_tracts + med_hh_inc_acs_10 + med_hh_inc_acs_10^2 + perc_green_tracts^2 + area_total_tracts^2 | 7 | 2759.78 | 48.88 | 0 | -1372.81 | 1 |
| BOS_18 | perc_green_tracts x popu_dens_tracts + perc_green_tracts^2 + popu_dens_tracts^2 | 7 | 2759.8 | 48.9 | 0 | -1372.82 | 1 |
| BOS_136 | area_total_tracts + white_pct_acs_10 + white_pct_acs_10^2 + perc_green_tracts^2 + area_total_tracts^2 | 7 | 2760.36 | 49.46 | 0 | -1373.1 | 1 |
| BOS_142 | perc_green_tracts + med_hh_inc_acs_10 + med_hh_inc_acs_10^2 + perc_green_tracts^2 | 6 | 2760.79 | 49.89 | 0 | -1374.34 | 1 |
| BOS_42 | perc_green_tracts + white_pct_acs_10 + white_pct_acs_10^2 | 5 | 2763.1 | 52.2 | 0 | -1376.51 | 1 |
| BOS_33 | perc_green_tracts + white_pct_acs_10 | 4 | 2765.2 | 54.3 | 0 | -1378.57 | 1 |
| BOS_5 | perc_green_tracts x area_total_tracts | 5 | 2765.87 | 54.97 | 0 | -1377.89 | 1 |
| BOS_17 | perc_green_tracts + popu_dens_tracts + perc_green_tracts^2 + popu_dens_tracts^2 | 6 | 2767.75 | 56.85 | 0 | -1377.82 | 1 |
| BOS_21 | area_total_tracts x popu_dens_tracts + perc_green_tracts^2 + area_total_tracts^2 + popu_dens_tracts^2 | 8 | 2768.44 | 57.55 | 0 | -1376.12 | 1 |
| BOS_51 | perc_green_tracts + med_hh_inc_acs_10 + med_hh_inc_acs_10^2 | 5 | 2768.63 | 57.74 | 0 | -1379.28 | 1 |
| BOS_4 | perc_green_tracts + area_total_tracts | 4 | 2771.93 | 61.03 | 0 | -1381.94 | 1 |
| BOS_68 | area_total_tracts x popu_dens_tracts + white_pct_acs_10 + med_hh_inc_acs_10 | 7 | 2775.86 | 64.96 | 0 | -1380.85 | 1 |
| BOS_113 | area_total_tracts x popu_dens_tracts + white_pct_acs_10 x med_hh_inc_acs_10 + med_hh_inc_acs_10^2 | 9 | 2776.2 | 65.3 | 0 | -1378.98 | 1 |
| BOS_104 | area_total_tracts x popu_dens_tracts + white_pct_acs_10 x med_hh_inc_acs_10 + white_pct_acs_10^2 | 9 | 2776.28 | 65.38 | 0 | -1379.02 | 1 |
| BOS_9 | perc_green_tracts x popu_dens_tracts | 5 | 2776.77 | 65.87 | 0 | -1383.34 | 1 |
| BOS_163 | white_pct_acs_10 + med_hh_inc_acs_10 + white_pct_acs_10^2 + perc_green_tracts^2 | 6 | 2777.25 | 66.35 | 0 | -1382.57 | 1 |
| BOS_86 | area_total_tracts x popu_dens_tracts + white_pct_acs_10 + med_hh_inc_acs_10 + med_hh_inc_acs_10^2 | 8 | 2777.31 | 66.41 | 0 | -1380.56 | 1 |
| BOS_8 | perc_green_tracts + popu_dens_tracts | 4 | 2777.54 | 66.64 | 0 | -1384.74 | 1 |
| BOS_193 | white_pct_acs_10 x med_hh_inc_acs_10 + white_pct_acs_10^2 + perc_green_tracts^2 | 7 | 2777.67 | 66.77 | 0 | -1381.76 | 1 |
| BOS_77 | area_total_tracts x popu_dens_tracts + white_pct_acs_10 + med_hh_inc_acs_10 + white_pct_acs_10^2 | 8 | 2777.86 | 66.96 | 0 | -1380.83 | 1 |
| BOS_183 | white_pct_acs_10 + med_hh_inc_acs_10 + white_pct_acs_10^2 + med_hh_inc_acs_10^2 + perc_green_tracts^2 | 7 | 2778.79 | 67.89 | 0 | -1382.32 | 1 |
| BOS_95 | area_total_tracts x popu_dens_tracts + white_pct_acs_10 + med_hh_inc_acs_10 + white_pct_acs_10^2 + med_hh_inc_acs_10^2 | 9 | 2779.32 | 68.42 | 0 | -1380.54 | 1 |
| BOS_213 | white_pct_acs_10 x med_hh_inc_acs_10 + white_pct_acs_10^2 + med_hh_inc_acs_10^2 + perc_green_tracts^2 | 8 | 2779.65 | 68.75 | 0 | -1381.73 | 1 |
| BOS_153 | white_pct_acs_10 + med_hh_inc_acs_10 + perc_green_tracts^2 | 5 | 2780.76 | 69.87 | 0 | -1385.34 | 1 |
| BOS_173 | white_pct_acs_10 + med_hh_inc_acs_10 + med_hh_inc_acs_10^2 + perc_green_tracts^2 | 6 | 2782.23 | 71.33 | 0 | -1385.06 | 1 |
| BOS_203 | white_pct_acs_10 x med_hh_inc_acs_10 + med_hh_inc_acs_10^2 + perc_green_tracts^2 | 7 | 2783.94 | 73.05 | 0 | -1384.89 | 1 |
| BOS_59 | area_total_tracts x popu_dens_tracts + med_hh_inc_acs_10 + med_hh_inc_acs_10^2 | 7 | 2787.54 | 76.64 | 0 | -1386.69 | 1 |
| BOS_16 | area_total_tracts + perc_green_tracts^2 + area_total_tracts^2 | 5 | 2789.79 | 78.9 | 0 | -1389.86 | 1 |
| BOS_41 | area_total_tracts x popu_dens_tracts + white_pct_acs_10 | 6 | 2792.36 | 81.47 | 0 | -1390.12 | 1 |
| BOS_99 | area_total_tracts + white_pct_acs_10 x med_hh_inc_acs_10 + white_pct_acs_10^2 | 7 | 2793.97 | 83.08 | 0 | -1389.91 | 1 |
| BOS_50 | area_total_tracts x popu_dens_tracts + white_pct_acs_10 + white_pct_acs_10^2 | 7 | 2794.38 | 83.48 | 0 | -1390.11 | 1 |
| BOS_117 | area_total_tracts + white_pct_acs_10 x med_hh_inc_acs_10 + white_pct_acs_10^2 + med_hh_inc_acs_10^2 | 8 | 2794.98 | 84.08 | 0 | -1389.39 | 1 |
| BOS_63 | area_total_tracts + white_pct_acs_10 + med_hh_inc_acs_10 | 5 | 2796.14 | 85.24 | 0 | -1393.03 | 1 |
| BOS_108 | area_total_tracts + white_pct_acs_10 x med_hh_inc_acs_10 + med_hh_inc_acs_10^2 | 7 | 2797.39 | 86.49 | 0 | -1391.62 | 1 |
| BOS_72 | area_total_tracts + white_pct_acs_10 + med_hh_inc_acs_10 + white_pct_acs_10^2 | 6 | 2797.65 | 86.75 | 0 | -1392.77 | 1 |
| BOS_81 | area_total_tracts + white_pct_acs_10 + med_hh_inc_acs_10 + med_hh_inc_acs_10^2 | 6 | 2798.17 | 87.27 | 0 | -1393.03 | 1 |
| BOS_90 | area_total_tracts + white_pct_acs_10 + med_hh_inc_acs_10 + white_pct_acs_10^2 + med_hh_inc_acs_10^2 | 7 | 2799.69 | 88.79 | 0 | -1392.77 | 1 |
| BOS_133 | white_pct_acs_10 + white_pct_acs_10^2 + perc_green_tracts^2 | 5 | 2800.47 | 89.58 | 0 | -1395.2 | 1 |
| BOS_13 | perc_green_tracts + perc_green_tracts^2 | 4 | 2803.98 | 93.09 | 0 | -1397.96 | 1 |
| BOS_123 | white_pct_acs_10 + perc_green_tracts^2 | 4 | 2805.16 | 94.26 | 0 | -1398.55 | 1 |
| BOS_143 | med_hh_inc_acs_10 + med_hh_inc_acs_10^2 + perc_green_tracts^2 | 5 | 2806.16 | 95.26 | 0 | -1398.04 | 1 |
| BOS_54 | area_total_tracts + med_hh_inc_acs_10 + med_hh_inc_acs_10^2 | 5 | 2813.6 | 102.71 | 0 | -1401.76 | 1 |
| BOS_12 | area_total_tracts x popu_dens_tracts | 5 | 2818.56 | 107.66 | 0 | -1404.24 | 1 |
| BOS_36 | area_total_tracts + white_pct_acs_10 | 4 | 2820.13 | 109.23 | 0 | -1406.04 | 1 |
| BOS_45 | area_total_tracts + white_pct_acs_10 + white_pct_acs_10^2 | 5 | 2821.56 | 110.66 | 0 | -1405.74 | 1 |
| BOS_3 | perc_green_tracts | 3 | 2823.91 | 113.01 | 0 | -1408.94 | 1 |
| BOS_30 | white_pct_acs_10 x med_hh_inc_acs_10 + white_pct_acs_10^2 | 6 | 2848.41 | 137.51 | 0 | -1418.15 | 1 |
| BOS_27 | white_pct_acs_10 + med_hh_inc_acs_10 + white_pct_acs_10^2 | 5 | 2849.03 | 138.13 | 0 | -1419.47 | 1 |
| BOS_32 | white_pct_acs_10 x med_hh_inc_acs_10 + white_pct_acs_10^2 + med_hh_inc_acs_10^2 | 7 | 2850.42 | 139.52 | 0 | -1418.13 | 1 |
| BOS_29 | white_pct_acs_10 + med_hh_inc_acs_10 + white_pct_acs_10^2 + med_hh_inc_acs_10^2 | 6 | 2850.87 | 139.97 | 0 | -1419.38 | 1 |
| BOS_26 | white_pct_acs_10 + med_hh_inc_acs_10 | 4 | 2853.4 | 142.5 | 0 | -1422.67 | 1 |
| BOS_28 | white_pct_acs_10 + med_hh_inc_acs_10 + med_hh_inc_acs_10^2 | 5 | 2855.19 | 144.29 | 0 | -1422.55 | 1 |
| BOS_31 | white_pct_acs_10 x med_hh_inc_acs_10 + med_hh_inc_acs_10^2 | 6 | 2857.13 | 146.24 | 0 | -1422.51 | 1 |
| BOS_6 | area_total_tracts | 3 | 2869.53 | 158.63 | 0 | -1431.75 | 1 |
| BOS_25 | med_hh_inc_acs_10 + med_hh_inc_acs_10^2 | 4 | 2883.89 | 173 | 0 | -1437.92 | 1 |
| BOS_7 | popu_dens_tracts | 3 | 2885.04 | 174.14 | 0 | -1439.5 | 1 |
| BOS_23 | white_pct_acs_10 + white_pct_acs_10^2 | 4 | 2887.51 | 176.61 | 0 | -1439.73 | 1 |
| BOS_24 | med_hh_inc_acs_10 | 3 | 2892.52 | 181.63 | 0 | -1443.25 | 1 |
| BOS_22 | white_pct_acs_10 | 3 | 2893.63 | 182.73 | 0 | -1443.8 | 1 |
| BOS_1 | 1 | 2 | 3001.15 | 290.25 | 0 | -1498.57 | 1 |
