# Supplementary material for: Evaluating the use of semi-structured crowdsourced data to quantify inequitable access to urban biodiversity: A case study with eBird
Source: PLoS One. 2022 Nov 9;17(11):e0277223. doi: 10.1371/journal.pone.0277223 (PMC9645630; doi:10.1371/journal.pone.0277223)
Supplement: S2 Table — (DOCX) [file pone.0277223.s002.docx]

**Table S2. Models included in the model averaging for PHX MSA.**Variable definitions: Prop_white = proportion of white residents, med_hh_income = median household income, prop_desert_tracts = proportion of tract that is desert, prop_cropland_tracts = proportion of tracts that is cropland, and prop_green_tracts = proportion of tract that is public green space, measured at the tract level. ^2 refers to a squared term. K = number of parameters, AICc = Corrected Akaike Information Criteria, Delta AICc = Difference from lowest AICc value, AICc Wt. = AICc weight, Log Lik. = Log Likelihood, Cum. Wt. = Cumulative Weight.

| **Model Name** | **Predictor Variables** | **K** | **AICc** | **Delta AICc** | **AICc Wt.** | **Log Lik.** | **Cum. Wt.** |
| --- | --- | --- | --- | --- | --- | --- | --- |
| PHX_77 | prop_desert_tracts x prop_cropland_tracts + prop_white + med_hh_income + prop_white^2+ x_coords + y_coords + x_coords^2 + y_coords^2 | 12 | 3039.94 | 0 | 0.13 | -1508 | 0.13 |
| PHX_75 | perc_green_tracts + prop_desert_tracts + prop_cropland_tracts + prop_white + med_hh_income + prop_white^2+ x_coords + y_coords + x_coords^2 + y_coords^2 | 12 | 3041.01 | 1.07 | 0.08 | -1508 | 0.21 |
| PHX_95 | prop_desert_tracts x prop_cropland_tracts + prop_white + med_hh_income + prop_white^2 + med_hh_income^2+ x_coords + y_coords + x_coords^2 + y_coords^2 | 13 | 3041.3 | 1.36 | 0.07 | -1507 | 0.28 |
| PHX_104 | prop_desert_tracts x prop_cropland_tracts + prop_white x med_hh_income + prop_white^2+ x_coords + y_coords + x_coords^2 + y_coords^2 | 13 | 3041.56 | 1.62 | 0.06 | -1508 | 0.34 |
| PHX_76 | perc_green_tracts + prop_desert_tracts x prop_cropland_tracts + prop_white + med_hh_income + prop_white^2+ x_coords + y_coords + x_coords^2 + y_coords^2 | 13 | 3042 | 2.06 | 0.05 | -1508 | 0.39 |
| PHX_93 | perc_green_tracts + prop_desert_tracts + prop_cropland_tracts + prop_white + med_hh_income + prop_white^2 + med_hh_income^2+ x_coords + y_coords + x_coords^2 + y_coords^2 | 13 | 3042.2 | 2.27 | 0.04 | -1508 | 0.43 |
| PHX_102 | perc_green_tracts + prop_desert_tracts + prop_cropland_tracts + prop_white x med_hh_income + prop_white^2+ x_coords + y_coords + x_coords^2 + y_coords^2 | 13 | 3042.64 | 2.7 | 0.03 | -1508 | 0.46 |
| PHX_169 | perc_green_tracts + prop_desert_tracts + prop_cropland_tracts + prop_white + med_hh_income + prop_white^2 + perc_green_tracts^2 + prop_desert_tracts^2 + prop_cropland_tracts^2+ x_coords + y_coords + x_coords^2 + y_coords^2 | 15 | 3042.87 | 2.94 | 0.03 | -1506 | 0.49 |
| PHX_120 | perc_green_tracts + prop_desert_tracts + prop_cropland_tracts + prop_white x med_hh_income + prop_white^2 + med_hh_income^2+ x_coords + y_coords + x_coords^2 + y_coords^2 | 14 | 3043.09 | 3.15 | 0.03 | -1507 | 0.52 |
| PHX_94 | perc_green_tracts + prop_desert_tracts x prop_cropland_tracts + prop_white + med_hh_income + prop_white^2 + med_hh_income^2+ x_coords + y_coords + x_coords^2 + y_coords^2 | 14 | 3043.37 | 3.43 | 0.02 | -1507 | 0.55 |
| PHX_165 | perc_green_tracts x prop_desert_tracts + prop_white + med_hh_income + prop_white^2 + perc_green_tracts^2 + prop_desert_tracts^2+ x_coords + y_coords + x_coords^2 + y_coords^2 | 14 | 3043.54 | 3.6 | 0.02 | -1508 | 0.57 |
| PHX_113 | prop_desert_tracts x prop_cropland_tracts + prop_white x med_hh_income + med_hh_income^2+ x_coords + y_coords + x_coords^2 + y_coords^2 | 13 | 3043.6 | 3.66 | 0.02 | -1509 | 0.59 |
| PHX_103 | perc_green_tracts + prop_desert_tracts x prop_cropland_tracts + prop_white x med_hh_income + prop_white^2+ x_coords + y_coords + x_coords^2 + y_coords^2 | 14 | 3043.62 | 3.69 | 0.02 | -1508 | 0.61 |
| PHX_68 | prop_desert_tracts x prop_cropland_tracts + prop_white + med_hh_income+ x_coords + y_coords + x_coords^2 + y_coords^2 | 11 | 3043.82 | 3.88 | 0.02 | -1511 | 0.63 |
| PHX_171 | prop_desert_tracts x prop_cropland_tracts + prop_white + med_hh_income + prop_white^2 + perc_green_tracts^2 + prop_desert_tracts^2 + prop_cropland_tracts^2+ x_coords + y_coords + x_coords^2 + y_coords^2 | 15 | 3044.02 | 4.08 | 0.02 | -1507 | 0.65 |
| PHX_185 | perc_green_tracts x prop_desert_tracts + prop_white + med_hh_income + prop_white^2 + med_hh_income^2 + perc_green_tracts^2 + prop_desert_tracts^2+ x_coords + y_coords + x_coords^2 + y_coords^2 | 15 | 3044.29 | 4.35 | 0.02 | -1507 | 0.66 |
| PHX_121 | perc_green_tracts + prop_desert_tracts x prop_cropland_tracts + prop_white x med_hh_income + prop_white^2 + med_hh_income^2+ x_coords + y_coords + x_coords^2 + y_coords^2 | 16 | 3044.31 | 4.38 | 0.01 | -1507 | 0.69 |
| PHX_189 | perc_green_tracts + prop_desert_tracts + prop_cropland_tracts + prop_white + med_hh_income + prop_white^2 + med_hh_income^2 + perc_green_tracts^2 + prop_desert_tracts^2 + prop_cropland_tracts^2+ x_coords + y_coords + x_coords^2 + y_coords^2 | 15 | 3044.31 | 4.38 | 0.01 | -1506 | 0.68 |
| PHX_170 | perc_green_tracts + prop_desert_tracts x prop_cropland_tracts + prop_white + med_hh_income + prop_white^2 + perc_green_tracts^2 + prop_desert_tracts^2 + prop_cropland_tracts^2+ x_coords + y_coords + x_coords^2 + y_coords^2 | 16 | 3044.4 | 4.47 | 0.01 | -1506 | 0.71 |
| PHX_111 | perc_green_tracts + prop_desert_tracts + prop_cropland_tracts + prop_white x med_hh_income + med_hh_income^2+ x_coords + y_coords + x_coords^2 + y_coords^2 | 13 | 3044.5 | 4.56 | 0.01 | -1509 | 0.72 |
| PHX_199 | perc_green_tracts + prop_desert_tracts + prop_cropland_tracts + prop_white x med_hh_income + prop_white^2 + perc_green_tracts^2 + prop_desert_tracts^2 + prop_cropland_tracts^2+ x_coords + y_coords + x_coords^2 + y_coords^2 | 16 | 3044.53 | 4.6 | 0.01 | -1506 | 0.73 |
| PHX_50 | prop_desert_tracts x prop_cropland_tracts + prop_white + prop_white^2+ x_coords + y_coords + x_coords^2 + y_coords^2 | 11 | 3044.86 | 4.92 | 0.01 | -1511 | 0.75 |
| PHX_66 | perc_green_tracts + prop_desert_tracts + prop_cropland_tracts + prop_white + med_hh_income+ x_coords + y_coords + x_coords^2 + y_coords^2 | 11 | 3045.01 | 5.07 | 0.01 | -1511 | 0.76 |
| PHX_72 | prop_desert_tracts + prop_white + med_hh_income + prop_white^2+ x_coords + y_coords + x_coords^2 + y_coords^2 | 10 | 3045.17 | 5.24 | 0.01 | -1512 | 0.77 |
| PHX_71 | perc_green_tracts x prop_desert_tracts + prop_white + med_hh_income + prop_white^2+ x_coords + y_coords + x_coords^2 + y_coords^2 | 12 | 3045.25 | 5.32 | 0.01 | -1510 | 0.78 |
| PHX_219 | perc_green_tracts + prop_desert_tracts + prop_cropland_tracts + prop_white x med_hh_income + prop_white^2 + med_hh_income^2 + perc_green_tracts^2 + prop_desert_tracts^2 + prop_cropland_tracts^2+ x_coords + y_coords + x_coords^2 + y_coords^2 | 17 | 3045.34 | 5.4 | 0.01 | -1505 | 0.78 |
| PHX_195 | perc_green_tracts x prop_desert_tracts + prop_white x med_hh_income + prop_white^2 + perc_green_tracts^2 + prop_desert_tracts^2+ x_coords + y_coords + x_coords^2 + y_coords^2 | 15 | 3045.44 | 5.5 | 0.01 | -1507 | 0.79 |
| PHX_86 | prop_desert_tracts x prop_cropland_tracts + prop_white + med_hh_income + med_hh_income^2+ x_coords + y_coords + x_coords^2 + y_coords^2 | 12 | 3045.46 | 5.52 | 0.01 | -1511 | 0.8 |
| PHX_48 | perc_green_tracts + prop_desert_tracts + prop_cropland_tracts + prop_white + prop_white^2+ x_coords + y_coords + x_coords^2 + y_coords^2 | 11 | 3045.48 | 5.54 | 0.01 | -1512 | 0.81 |
| PHX_215 | perc_green_tracts x prop_desert_tracts + prop_white x med_hh_income + prop_white^2 + med_hh_income^2 + perc_green_tracts^2 + prop_desert_tracts^2+ x_coords + y_coords + x_coords^2 + y_coords^2 | 16 | 3045.51 | 5.57 | 0.01 | -1506 | 0.82 |
| PHX_191 | prop_desert_tracts x prop_cropland_tracts + prop_white + med_hh_income + prop_white^2 + med_hh_income^2 + perc_green_tracts^2 + prop_desert_tracts^2 + prop_cropland_tracts^2+ x_coords + y_coords + x_coords^2 + y_coords^2 | 16 | 3045.53 | 5.59 | 0.01 | -1506 | 0.83 |
| PHX_112 | perc_green_tracts + prop_desert_tracts x prop_cropland_tracts + prop_white x med_hh_income + med_hh_income^2+ x_coords + y_coords + x_coords^2 + y_coords^2 | 14 | 3045.66 | 5.72 | 0.01 | -1509 | 0.83 |
| PHX_201 | prop_desert_tracts x prop_cropland_tracts + prop_white x med_hh_income + prop_white^2 + perc_green_tracts^2 + prop_desert_tracts^2 + prop_cropland_tracts^2+ x_coords + y_coords + x_coords^2 + y_coords^2 | 16 | 3045.66 | 5.72 | 0.01 | -1507 | 0.84 |
| PHX_90 | prop_desert_tracts + prop_white + med_hh_income + prop_white^2 + med_hh_income^2+ x_coords + y_coords + x_coords^2 + y_coords^2 | 11 | 3045.79 | 5.85 | 0.01 | -1512 | 0.85 |
| PHX_67 | perc_green_tracts + prop_desert_tracts x prop_cropland_tracts + prop_white + med_hh_income+ x_coords + y_coords + x_coords^2 + y_coords^2 | 12 | 3045.87 | 5.93 | 0.01 | -1511 | 0.86 |
| PHX_89 | perc_green_tracts x prop_desert_tracts + prop_white + med_hh_income + prop_white^2 + med_hh_income^2+ x_coords + y_coords + x_coords^2 + y_coords^2 | 13 | 3045.88 | 5.94 | 0.01 | -1510 | 0.86 |
| PHX_190 | perc_green_tracts + prop_desert_tracts x prop_cropland_tracts + prop_white + med_hh_income + prop_white^2 + med_hh_income^2 + perc_green_tracts^2 + prop_desert_tracts^2 + prop_cropland_tracts^2+ x_coords + y_coords + x_coords^2 + y_coords^2 | 17 | 3045.93 | 6 | 0.01 | -1506 | 0.87 |
| PHX_200 | perc_green_tracts + prop_desert_tracts x prop_cropland_tracts + prop_white x med_hh_income + prop_white^2 + perc_green_tracts^2 + prop_desert_tracts^2 + prop_cropland_tracts^2+ x_coords + y_coords + x_coords^2 + y_coords^2 | 17 | 3046.05 | 6.12 | 0.01 | -1506 | 0.88 |
| PHX_159 | perc_green_tracts + prop_desert_tracts + prop_cropland_tracts + prop_white + med_hh_income + perc_green_tracts^2 + prop_desert_tracts^2 + prop_cropland_tracts^2+ x_coords + y_coords + x_coords^2 + y_coords^2 | 14 | 3046.22 | 6.28 | 0.01 | -1509 | 0.88 |
| PHX_139 | perc_green_tracts + prop_desert_tracts + prop_cropland_tracts + prop_white + prop_white^2 + perc_green_tracts^2 + prop_desert_tracts^2 + prop_cropland_tracts^2+ x_coords + y_coords + x_coords^2 + y_coords^2 | 14 | 3046.25 | 6.31 | 0.01 | -1509 | 0.89 |
| PHX_155 | perc_green_tracts x prop_desert_tracts + prop_white + med_hh_income + perc_green_tracts^2 + prop_desert_tracts^2+ x_coords + y_coords + x_coords^2 + y_coords^2 | 13 | 3046.32 | 6.38 | 0.01 | -1510 | 0.89 |
| PHX_205 | perc_green_tracts x prop_desert_tracts + prop_white x med_hh_income + med_hh_income^2 + perc_green_tracts^2 + prop_desert_tracts^2+ x_coords + y_coords + x_coords^2 + y_coords^2 | 15 | 3046.38 | 6.44 | 0.01 | -1508 | 0.9 |
| PHX_209 | perc_green_tracts + prop_desert_tracts + prop_cropland_tracts + prop_white x med_hh_income + med_hh_income^2 + perc_green_tracts^2 + prop_desert_tracts^2 + prop_cropland_tracts^2+ x_coords + y_coords + x_coords^2 + y_coords^2 | 16 | 3046.38 | 6.44 | 0.01 | -1507 | 0.9 |
| PHX_84 | perc_green_tracts + prop_desert_tracts + prop_cropland_tracts + prop_white + med_hh_income + med_hh_income^2+ x_coords + y_coords + x_coords^2 + y_coords^2 | 12 | 3046.52 | 6.58 | 0 | -1511 | 0.91 |
| PHX_2 | prop_white x med_hh_income + prop_white^2 + med_hh_income^2 + prop_desert_tracts x prop_cropland_tracts + perc_green_tracts^2 + prop_desert_tracts^2 + prop_cropland_tracts^2+ x_coords + y_coords + x_coords^2 + y_coords^2 | 17 | 3046.55 | 6.62 | 0 | -1506 | 0.91 |
| PHX_117 | prop_desert_tracts + prop_white x med_hh_income + prop_white^2 + med_hh_income^2+ x_coords + y_coords + x_coords^2 + y_coords^2 | 12 | 3046.88 | 6.95 | 0 | -1511 | 0.92 |
| PHX_49 | perc_green_tracts + prop_desert_tracts x prop_cropland_tracts + prop_white + prop_white^2+ x_coords + y_coords + x_coords^2 + y_coords^2 | 12 | 3046.92 | 6.98 | 0 | -1511 | 0.92 |
| PHX_116 | perc_green_tracts x prop_desert_tracts + prop_white x med_hh_income + prop_white^2 + med_hh_income^2+ x_coords + y_coords + x_coords^2 + y_coords^2 | 14 | 3046.96 | 7.02 | 0 | -1509 | 0.92 |
| PHX_220 | perc_green_tracts + prop_desert_tracts x prop_cropland_tracts + prop_white x med_hh_income + prop_white^2 + med_hh_income^2 + perc_green_tracts^2 + prop_desert_tracts^2 + prop_cropland_tracts^2+ x_coords + y_coords + x_coords^2 + y_coords^2 | 18 | 3046.99 | 7.06 | 0 | -1505 | 0.93 |
| PHX_99 | prop_desert_tracts + prop_white x med_hh_income + prop_white^2+ x_coords + y_coords + x_coords^2 + y_coords^2 | 11 | 3047.03 | 7.09 | 0 | -1512 | 0.93 |
| PHX_98 | perc_green_tracts x prop_desert_tracts + prop_white x med_hh_income + prop_white^2+ x_coords + y_coords + x_coords^2 + y_coords^2 | 13 | 3047.11 | 7.17 | 0 | -1510 | 0.94 |
| PHX_70 | perc_green_tracts + prop_desert_tracts + prop_white + med_hh_income + prop_white^2+ x_coords + y_coords + x_coords^2 + y_coords^2 | 11 | 3047.21 | 7.27 | 0 | -1512 | 0.94 |
| PHX_175 | perc_green_tracts x prop_desert_tracts + prop_white + med_hh_income + med_hh_income^2 + perc_green_tracts^2 + prop_desert_tracts^2+ x_coords + y_coords + x_coords^2 + y_coords^2 | 14 | 3047.45 | 7.51 | 0 | -1509 | 0.94 |
| PHX_85 | perc_green_tracts + prop_desert_tracts x prop_cropland_tracts + prop_white + med_hh_income + med_hh_income^2+ x_coords + y_coords + x_coords^2 + y_coords^2 | 13 | 3047.52 | 7.58 | 0 | -1511 | 0.95 |
| PHX_160 | perc_green_tracts + prop_desert_tracts x prop_cropland_tracts + prop_white + med_hh_income + perc_green_tracts^2 + prop_desert_tracts^2 + prop_cropland_tracts^2+ x_coords + y_coords + x_coords^2 + y_coords^2 | 15 | 3047.69 | 7.76 | 0 | -1509 | 0.95 |
| PHX_161 | prop_desert_tracts x prop_cropland_tracts + prop_white + med_hh_income + perc_green_tracts^2 + prop_desert_tracts^2 + prop_cropland_tracts^2+ x_coords + y_coords + x_coords^2 + y_coords^2 | 14 | 3047.77 | 7.83 | 0 | -1510 | 0.95 |
| PHX_88 | perc_green_tracts + prop_desert_tracts + prop_white + med_hh_income + prop_white^2 + med_hh_income^2+ x_coords + y_coords + x_coords^2 + y_coords^2 | 12 | 3047.84 | 7.9 | 0 | -1512 | 0.95 |
| PHX_211 | prop_desert_tracts x prop_cropland_tracts + prop_white x med_hh_income + med_hh_income^2 + perc_green_tracts^2 + prop_desert_tracts^2 + prop_cropland_tracts^2+ x_coords + y_coords + x_coords^2 + y_coords^2 | 16 | 3047.85 | 7.92 | 0 | -1508 | 0.96 |
| PHX_179 | perc_green_tracts + prop_desert_tracts + prop_cropland_tracts + prop_white + med_hh_income + med_hh_income^2 + perc_green_tracts^2 + prop_desert_tracts^2 + prop_cropland_tracts^2+ x_coords + y_coords + x_coords^2 + y_coords^2 | 15 | 3047.92 | 7.98 | 0 | -1509 | 0.96 |
| PHX_210 | perc_green_tracts + prop_desert_tracts x prop_cropland_tracts + prop_white x med_hh_income + med_hh_income^2 + perc_green_tracts^2 + prop_desert_tracts^2 + prop_cropland_tracts^2+ x_coords + y_coords + x_coords^2 + y_coords^2 | 17 | 3048 | 8.06 | 0 | -1507 | 0.96 |
| PHX_141 | prop_desert_tracts x prop_cropland_tracts + prop_white + prop_white^2 + perc_green_tracts^2 + prop_desert_tracts^2 + prop_cropland_tracts^2+ x_coords + y_coords + x_coords^2 + y_coords^2 | 14 | 3048.02 | 8.08 | 0 | -1510 | 0.96 |
| PHX_108 | prop_desert_tracts + prop_white x med_hh_income + med_hh_income^2+ x_coords + y_coords + x_coords^2 + y_coords^2 | 11 | 3048.09 | 8.15 | 0 | -1513 | 0.97 |
| PHX_140 | perc_green_tracts + prop_desert_tracts x prop_cropland_tracts + prop_white + prop_white^2 + perc_green_tracts^2 + prop_desert_tracts^2 + prop_cropland_tracts^2+ x_coords + y_coords + x_coords^2 + y_coords^2 | 15 | 3048.13 | 8.19 | 0 | -1509 | 0.97 |
| PHX_107 | perc_green_tracts x prop_desert_tracts + prop_white x med_hh_income + med_hh_income^2+ x_coords + y_coords + x_coords^2 + y_coords^2 | 13 | 3048.3 | 8.37 | 0 | -1511 | 0.97 |
| PHX_63 | prop_desert_tracts + prop_white + med_hh_income+ x_coords + y_coords + x_coords^2 + y_coords^2 | 9 | 3048.54 | 8.6 | 0 | -1515 | 0.97 |
| PHX_166 | prop_desert_tracts + prop_white + med_hh_income + prop_white^2 + perc_green_tracts^2 + prop_desert_tracts^2+ x_coords + y_coords + x_coords^2 + y_coords^2 | 12 | 3048.6 | 8.66 | 0 | -1512 | 0.97 |
| PHX_164 | perc_green_tracts + prop_desert_tracts + prop_white + med_hh_income + prop_white^2 + perc_green_tracts^2 + prop_desert_tracts^2+ x_coords + y_coords + x_coords^2 + y_coords^2 | 13 | 3048.73 | 8.8 | 0 | -1511 | 0.98 |
| PHX_62 | perc_green_tracts x prop_desert_tracts + prop_white + med_hh_income+ x_coords + y_coords + x_coords^2 + y_coords^2 | 11 | 3048.84 | 8.9 | 0 | -1513 | 0.98 |
| PHX_115 | perc_green_tracts + prop_desert_tracts + prop_white x med_hh_income + prop_white^2 + med_hh_income^2+ x_coords + y_coords + x_coords^2 + y_coords^2 | 13 | 3048.93 | 9 | 0 | -1511 | 0.98 |
| PHX_41 | prop_desert_tracts x prop_cropland_tracts + prop_white+ x_coords + y_coords + x_coords^2 + y_coords^2 | 10 | 3048.94 | 9 | 0 | -1514 | 0.98 |
| PHX_97 | perc_green_tracts + prop_desert_tracts + prop_white x med_hh_income + prop_white^2+ x_coords + y_coords + x_coords^2 + y_coords^2 | 12 | 3049.06 | 9.13 | 0 | -1512 | 0.98 |
| PHX_186 | prop_desert_tracts + prop_white + med_hh_income + prop_white^2 + med_hh_income^2 + perc_green_tracts^2 + prop_desert_tracts^2+ x_coords + y_coords + x_coords^2 + y_coords^2 | 13 | 3049.29 | 9.35 | 0 | -1511 | 0.98 |
| PHX_180 | perc_green_tracts + prop_desert_tracts x prop_cropland_tracts + prop_white + med_hh_income + med_hh_income^2 + perc_green_tracts^2 + prop_desert_tracts^2 + prop_cropland_tracts^2+ x_coords + y_coords + x_coords^2 + y_coords^2 | 16 | 3049.46 | 9.53 | 0 | -1508 | 0.98 |
| PHX_184 | perc_green_tracts + prop_desert_tracts + prop_white + med_hh_income + prop_white^2 + med_hh_income^2 + perc_green_tracts^2 + prop_desert_tracts^2+ x_coords + y_coords + x_coords^2 + y_coords^2 | 14 | 3049.47 | 9.54 | 0 | -1510 | 0.99 |
| PHX_181 | prop_desert_tracts x prop_cropland_tracts + prop_white + med_hh_income + med_hh_income^2 + perc_green_tracts^2 + prop_desert_tracts^2 + prop_cropland_tracts^2+ x_coords + y_coords + x_coords^2 + y_coords^2 | 15 | 3049.52 | 9.59 | 0 | -1509 | 0.99 |
| PHX_81 | prop_desert_tracts + prop_white + med_hh_income + med_hh_income^2+ x_coords + y_coords + x_coords^2 + y_coords^2 | 10 | 3049.57 | 9.63 | 0 | -1515 | 0.99 |
| PHX_39 | perc_green_tracts + prop_desert_tracts + prop_cropland_tracts + prop_white+ x_coords + y_coords + x_coords^2 + y_coords^2 | 10 | 3049.65 | 9.71 | 0 | -1515 | 0.99 |
| PHX_129 | perc_green_tracts + prop_desert_tracts + prop_cropland_tracts + prop_white + perc_green_tracts^2 + prop_desert_tracts^2 + prop_cropland_tracts^2+ x_coords + y_coords + x_coords^2 + y_coords^2 | 13 | 3049.67 | 9.73 | 0 | -1512 | 0.99 |
| PHX_80 | perc_green_tracts x prop_desert_tracts + prop_white + med_hh_income + med_hh_income^2+ x_coords + y_coords + x_coords^2 + y_coords^2 | 12 | 3049.89 | 9.95 | 0 | -1513 | 0.99 |
| PHX_106 | perc_green_tracts + prop_desert_tracts + prop_white x med_hh_income + med_hh_income^2+ x_coords + y_coords + x_coords^2 + y_coords^2 | 12 | 3050.12 | 10.18 | 0 | -1513 | 0.99 |
| PHX_216 | prop_desert_tracts + prop_white x med_hh_income + prop_white^2 + med_hh_income^2 + perc_green_tracts^2 + prop_desert_tracts^2+ x_coords + y_coords + x_coords^2 + y_coords^2 | 14 | 3050.47 | 10.53 | 0 | -1511 | 0.99 |
| PHX_196 | prop_desert_tracts + prop_white x med_hh_income + prop_white^2 + perc_green_tracts^2 + prop_desert_tracts^2+ x_coords + y_coords + x_coords^2 + y_coords^2 | 13 | 3050.48 | 10.55 | 0 | -1512 | 0.99 |
| PHX_61 | perc_green_tracts + prop_desert_tracts + prop_white + med_hh_income+ x_coords + y_coords + x_coords^2 + y_coords^2 | 10 | 3050.56 | 10.63 | 0 | -1515 | 0.99 |
| PHX_194 | perc_green_tracts + prop_desert_tracts + prop_white x med_hh_income + prop_white^2 + perc_green_tracts^2 + prop_desert_tracts^2+ x_coords + y_coords + x_coords^2 + y_coords^2 | 14 | 3050.63 | 10.69 | 0 | -1511 | 0.99 |
| PHX_214 | perc_green_tracts + prop_desert_tracts + prop_white x med_hh_income + prop_white^2 + med_hh_income^2 + perc_green_tracts^2 + prop_desert_tracts^2+ x_coords + y_coords + x_coords^2 + y_coords^2 | 15 | 3050.68 | 10.75 | 0 | -1510 | 0.99 |
| PHX_40 | perc_green_tracts + prop_desert_tracts x prop_cropland_tracts + prop_white+ x_coords + y_coords + x_coords^2 + y_coords^2 | 11 | 3050.99 | 11.05 | 0 | -1514 | 0.99 |
| PHX_135 | perc_green_tracts x prop_desert_tracts + prop_white + prop_white^2 + perc_green_tracts^2 + prop_desert_tracts^2+ x_coords + y_coords + x_coords^2 + y_coords^2 | 13 | 3051.02 | 11.08 | 0 | -1512 | 1 |
| PHX_154 | perc_green_tracts + prop_desert_tracts + prop_white + med_hh_income + perc_green_tracts^2 + prop_desert_tracts^2+ x_coords + y_coords + x_coords^2 + y_coords^2 | 12 | 3051.5 | 11.56 | 0 | -1514 | 1 |
| PHX_130 | perc_green_tracts + prop_desert_tracts x prop_cropland_tracts + prop_white + perc_green_tracts^2 + prop_desert_tracts^2 + prop_cropland_tracts^2+ x_coords + y_coords + x_coords^2 + y_coords^2 | 14 | 3051.51 | 11.57 | 0 | -1512 | 1 |
| PHX_204 | perc_green_tracts + prop_desert_tracts + prop_white x med_hh_income + med_hh_income^2 + perc_green_tracts^2 + prop_desert_tracts^2+ x_coords + y_coords + x_coords^2 + y_coords^2 | 14 | 3051.54 | 11.6 | 0 | -1512 | 1 |
| PHX_79 | perc_green_tracts + prop_desert_tracts + prop_white + med_hh_income + med_hh_income^2+ x_coords + y_coords + x_coords^2 + y_coords^2 | 11 | 3051.6 | 11.67 | 0 | -1515 | 1 |
| PHX_206 | prop_desert_tracts + prop_white x med_hh_income + med_hh_income^2 + perc_green_tracts^2 + prop_desert_tracts^2+ x_coords + y_coords + x_coords^2 + y_coords^2 | 13 | 3051.63 | 11.69 | 0 | -1513 | 1 |
| PHX_156 | prop_desert_tracts + prop_white + med_hh_income + perc_green_tracts^2 + prop_desert_tracts^2+ x_coords + y_coords + x_coords^2 + y_coords^2 | 11 | 3051.82 | 11.88 | 0 | -1515 | 1 |
| PHX_131 | prop_desert_tracts x prop_cropland_tracts + prop_white + perc_green_tracts^2 + prop_desert_tracts^2 + prop_cropland_tracts^2+ x_coords + y_coords + x_coords^2 + y_coords^2 | 13 | 3051.9 | 11.96 | 0 | -1513 | 1 |
| PHX_174 | perc_green_tracts + prop_desert_tracts + prop_white + med_hh_income + med_hh_income^2 + perc_green_tracts^2 + prop_desert_tracts^2+ x_coords + y_coords + x_coords^2 + y_coords^2 | 13 | 3052.63 | 12.69 | 0 | -1513 | 1 |
| PHX_176 | prop_desert_tracts + prop_white + med_hh_income + med_hh_income^2 + perc_green_tracts^2 + prop_desert_tracts^2+ x_coords + y_coords + x_coords^2 + y_coords^2 | 12 | 3052.91 | 12.98 | 0 | -1514 | 1 |
| PHX_125 | perc_green_tracts x prop_desert_tracts + prop_white + perc_green_tracts^2 + prop_desert_tracts^2+ x_coords + y_coords + x_coords^2 + y_coords^2 | 12 | 3053.72 | 13.78 | 0 | -1515 | 1 |
| PHX_167 | perc_green_tracts + prop_cropland_tracts + prop_white + med_hh_income + prop_white^2 + perc_green_tracts^2 + prop_cropland_tracts^2+ x_coords + y_coords + x_coords^2 + y_coords^2 | 13 | 3053.87 | 13.93 | 0 | -1514 | 1 |
| PHX_44 | perc_green_tracts x prop_desert_tracts + prop_white + prop_white^2+ x_coords + y_coords + x_coords^2 + y_coords^2 | 11 | 3054.36 | 14.42 | 0 | -1516 | 1 |
| PHX_45 | prop_desert_tracts + prop_white + prop_white^2+ x_coords + y_coords + x_coords^2 + y_coords^2 | 9 | 3054.46 | 14.52 | 0 | -1518 | 1 |
| PHX_73 | perc_green_tracts + prop_cropland_tracts + prop_white + med_hh_income + prop_white^2+ x_coords + y_coords + x_coords^2 + y_coords^2 | 11 | 3054.57 | 14.64 | 0 | -1516 | 1 |
| PHX_197 | perc_green_tracts + prop_cropland_tracts + prop_white x med_hh_income + prop_white^2 + perc_green_tracts^2 + prop_cropland_tracts^2+ x_coords + y_coords + x_coords^2 + y_coords^2 | 14 | 3054.93 | 14.99 | 0 | -1513 | 1 |
| PHX_100 | perc_green_tracts + prop_cropland_tracts + prop_white x med_hh_income + prop_white^2+ x_coords + y_coords + x_coords^2 + y_coords^2 | 12 | 3055.61 | 15.68 | 0 | -1516 | 1 |
| PHX_168 | perc_green_tracts x prop_cropland_tracts + prop_white + med_hh_income + prop_white^2 + perc_green_tracts^2 + prop_cropland_tracts^2+ x_coords + y_coords + x_coords^2 + y_coords^2 | 14 | 3055.73 | 15.79 | 0 | -1514 | 1 |
| PHX_187 | perc_green_tracts + prop_cropland_tracts + prop_white + med_hh_income + prop_white^2 + med_hh_income^2 + perc_green_tracts^2 + prop_cropland_tracts^2+ x_coords + y_coords + x_coords^2 + y_coords^2 | 14 | 3055.86 | 15.92 | 0 | -1514 | 1 |
| PHX_74 | perc_green_tracts x prop_cropland_tracts + prop_white + med_hh_income + prop_white^2+ x_coords + y_coords + x_coords^2 + y_coords^2 | 12 | 3056.08 | 16.15 | 0 | -1516 | 1 |
| PHX_91 | perc_green_tracts + prop_cropland_tracts + prop_white + med_hh_income + prop_white^2 + med_hh_income^2+ x_coords + y_coords + x_coords^2 + y_coords^2 | 15 | 3056.48 | 16.55 | 0 | -1516 | 1 |
| PHX_217 | perc_green_tracts + prop_cropland_tracts + prop_white x med_hh_income + prop_white^2 + med_hh_income^2 + perc_green_tracts^2 + prop_cropland_tracts^2+ x_coords + y_coords + x_coords^2 + y_coords^2 | 12 | 3056.48 | 16.54 | 0 | -1513 | 1 |
| PHX_43 | perc_green_tracts + prop_desert_tracts + prop_white + prop_white^2+ x_coords + y_coords + x_coords^2 + y_coords^2 | 10 | 3056.49 | 16.56 | 0 | -1518 | 1 |
| PHX_157 | perc_green_tracts + prop_cropland_tracts + prop_white + med_hh_income + perc_green_tracts^2 + prop_cropland_tracts^2+ x_coords + y_coords + x_coords^2 + y_coords^2 | 12 | 3056.59 | 16.66 | 0 | -1516 | 1 |
| PHX_207 | perc_green_tracts + prop_cropland_tracts + prop_white x med_hh_income + med_hh_income^2 + perc_green_tracts^2 + prop_cropland_tracts^2+ x_coords + y_coords + x_coords^2 + y_coords^2 | 14 | 3056.75 | 16.81 | 0 | -1514 | 1 |
| PHX_198 | perc_green_tracts x prop_cropland_tracts + prop_white x med_hh_income + prop_white^2 + perc_green_tracts^2 + prop_cropland_tracts^2+ x_coords + y_coords + x_coords^2 + y_coords^2 | 15 | 3056.77 | 16.84 | 0 | -1513 | 1 |
| PHX_134 | perc_green_tracts + prop_desert_tracts + prop_white + prop_white^2 + perc_green_tracts^2 + prop_desert_tracts^2+ x_coords + y_coords + x_coords^2 + y_coords^2 | 12 | 3056.82 | 16.88 | 0 | -1516 | 1 |
| PHX_118 | perc_green_tracts + prop_cropland_tracts + prop_white x med_hh_income + prop_white^2 + med_hh_income^2+ x_coords + y_coords + x_coords^2 + y_coords^2 | 13 | 3056.97 | 17.03 | 0 | -1515 | 1 |
| PHX_101 | perc_green_tracts x prop_cropland_tracts + prop_white x med_hh_income + prop_white^2+ x_coords + y_coords + x_coords^2 + y_coords^2 | 13 | 3057.09 | 17.15 | 0 | -1515 | 1 |
| PHX_136 | prop_desert_tracts + prop_white + prop_white^2 + perc_green_tracts^2 + prop_desert_tracts^2+ x_coords + y_coords + x_coords^2 + y_coords^2 | 11 | 3057.15 | 17.22 | 0 | -1517 | 1 |
| PHX_27 | prop_white + med_hh_income + prop_white^2+ x_coords + y_coords + x_coords^2 + y_coords^2 | 9 | 3057.36 | 17.43 | 0 | -1520 | 1 |
| PHX_109 | perc_green_tracts + prop_cropland_tracts + prop_white x med_hh_income + med_hh_income^2+ x_coords + y_coords + x_coords^2 + y_coords^2 | 12 | 3057.56 | 17.62 | 0 | -1517 | 1 |
| PHX_188 | perc_green_tracts x prop_cropland_tracts + prop_white + med_hh_income + prop_white^2 + med_hh_income^2 + perc_green_tracts^2 + prop_cropland_tracts^2+ x_coords + y_coords + x_coords^2 + y_coords^2 | 15 | 3057.73 | 17.79 | 0 | -1514 | 1 |
| PHX_36 | prop_desert_tracts + prop_white+ x_coords + y_coords + x_coords^2 + y_coords^2 | 8 | 3057.85 | 17.91 | 0 | -1521 | 1 |
| PHX_64 | perc_green_tracts + prop_cropland_tracts + prop_white + med_hh_income+ x_coords + y_coords + x_coords^2 + y_coords^2 | 10 | 3057.88 | 17.95 | 0 | -1519 | 1 |
| PHX_35 | perc_green_tracts x prop_desert_tracts + prop_white+ x_coords + y_coords + x_coords^2 + y_coords^2 | 10 | 3057.97 | 18.03 | 0 | -1519 | 1 |
| PHX_92 | perc_green_tracts x prop_cropland_tracts + prop_white + med_hh_income + prop_white^2 + med_hh_income^2+ x_coords + y_coords + x_coords^2 + y_coords^2 | 13 | 3058 | 18.06 | 0 | -1516 | 1 |
| PHX_218 | perc_green_tracts x prop_cropland_tracts + prop_white x med_hh_income + prop_white^2 + med_hh_income^2 + perc_green_tracts^2 + prop_cropland_tracts^2+ x_coords + y_coords + x_coords^2 + y_coords^2 | 16 | 3058.32 | 18.39 | 0 | -1513 | 1 |
| PHX_119 | perc_green_tracts x prop_cropland_tracts + prop_white x med_hh_income + prop_white^2 + med_hh_income^2+ x_coords + y_coords + x_coords^2 + y_coords^2 | 14 | 3058.45 | 18.51 | 0 | -1515 | 1 |
| PHX_158 | perc_green_tracts x prop_cropland_tracts + prop_white + med_hh_income + perc_green_tracts^2 + prop_cropland_tracts^2+ x_coords + y_coords + x_coords^2 + y_coords^2 | 13 | 3058.48 | 18.54 | 0 | -1516 | 1 |
| PHX_208 | perc_green_tracts x prop_cropland_tracts + prop_white x med_hh_income + med_hh_income^2 + perc_green_tracts^2 + prop_cropland_tracts^2+ x_coords + y_coords + x_coords^2 + y_coords^2 | 15 | 3058.59 | 18.65 | 0 | -1514 | 1 |
| PHX_177 | perc_green_tracts + prop_cropland_tracts + prop_white + med_hh_income + med_hh_income^2 + perc_green_tracts^2 + prop_cropland_tracts^2+ x_coords + y_coords + x_coords^2 + y_coords^2 | 13 | 3058.64 | 18.7 | 0 | -1516 | 1 |
| PHX_162 | perc_green_tracts + prop_white + med_hh_income + prop_white^2 + perc_green_tracts^2+ x_coords + y_coords + x_coords^2 + y_coords^2 | 11 | 3058.77 | 18.83 | 0 | -1518 | 1 |
| PHX_30 | prop_white x med_hh_income + prop_white^2+ x_coords + y_coords + x_coords^2 + y_coords^2 | 10 | 3058.78 | 18.84 | 0 | -1519 | 1 |
| PHX_29 | prop_white + med_hh_income + prop_white^2 + med_hh_income^2+ x_coords + y_coords + x_coords^2 + y_coords^2 | 10 | 3058.98 | 19.04 | 0 | -1519 | 1 |
| PHX_110 | perc_green_tracts x prop_cropland_tracts + prop_white x med_hh_income + med_hh_income^2+ x_coords + y_coords + x_coords^2 + y_coords^2 | 13 | 3059.01 | 19.07 | 0 | -1516 | 1 |
| PHX_69 | perc_green_tracts + prop_white + med_hh_income + prop_white^2+ x_coords + y_coords + x_coords^2 + y_coords^2 | 10 | 3059.3 | 19.36 | 0 | -1520 | 1 |
| PHX_65 | perc_green_tracts x prop_cropland_tracts + prop_white + med_hh_income+ x_coords + y_coords + x_coords^2 + y_coords^2 | 10 | 3059.4 | 19.46 | 0 | -1519 | 1 |
| PHX_163 | prop_white + med_hh_income + prop_white^2 + perc_green_tracts^2+ x_coords + y_coords + x_coords^2 + y_coords^2 | 11 | 3059.4 | 19.46 | 0 | -1520 | 1 |
| PHX_124 | perc_green_tracts + prop_desert_tracts + prop_white + perc_green_tracts^2 + prop_desert_tracts^2+ x_coords + y_coords + x_coords^2 + y_coords^2 | 11 | 3059.51 | 19.57 | 0 | -1519 | 1 |
| PHX_32 | prop_white x med_hh_income + prop_white^2 + med_hh_income^2+ x_coords + y_coords + x_coords^2 + y_coords^2 | 11 | 3059.72 | 19.78 | 0 | -1519 | 1 |
| PHX_34 | perc_green_tracts + prop_desert_tracts + prop_white+ x_coords + y_coords + x_coords^2 + y_coords^2 | 9 | 3059.87 | 19.93 | 0 | -1521 | 1 |
| PHX_82 | perc_green_tracts + prop_cropland_tracts + prop_white + med_hh_income + med_hh_income^2+ x_coords + y_coords + x_coords^2 + y_coords^2 | 11 | 3059.89 | 19.95 | 0 | -1519 | 1 |
| PHX_26 | prop_white + med_hh_income+ x_coords + y_coords + x_coords^2 + y_coords^2 | 8 | 3060.17 | 20.24 | 0 | -1522 | 1 |
| PHX_31 | prop_white x med_hh_income + med_hh_income^2+ x_coords + y_coords + x_coords^2 + y_coords^2 | 10 | 3060.18 | 20.24 | 0 | -1520 | 1 |
| PHX_192 | perc_green_tracts + prop_white x med_hh_income + prop_white^2 + perc_green_tracts^2+ x_coords + y_coords + x_coords^2 + y_coords^2 | 12 | 3060.22 | 20.28 | 0 | -1518 | 1 |
| PHX_126 | prop_desert_tracts + prop_white + perc_green_tracts^2 + prop_desert_tracts^2+ x_coords + y_coords + x_coords^2 + y_coords^2 | 10 | 3060.34 | 20.4 | 0 | -1520 | 1 |
| PHX_182 | perc_green_tracts + prop_white + med_hh_income + prop_white^2 + med_hh_income^2 + perc_green_tracts^2+ x_coords + y_coords + x_coords^2 + y_coords^2 | 12 | 3060.4 | 20.46 | 0 | -1518 | 1 |
| PHX_178 | perc_green_tracts x prop_cropland_tracts + prop_white + med_hh_income + med_hh_income^2 + perc_green_tracts^2 + prop_cropland_tracts^2+ x_coords + y_coords + x_coords^2 + y_coords^2 | 14 | 3060.53 | 20.59 | 0 | -1516 | 1 |
| PHX_96 | perc_green_tracts + prop_white x med_hh_income + prop_white^2+ x_coords + y_coords + x_coords^2 + y_coords^2 | 11 | 3060.7 | 20.76 | 0 | -1519 | 1 |
| PHX_193 | prop_white x med_hh_income + prop_white^2 + perc_green_tracts^2+ x_coords + y_coords + x_coords^2 + y_coords^2 | 11 | 3060.82 | 20.88 | 0 | -1519 | 1 |
| PHX_87 | perc_green_tracts + prop_white + med_hh_income + prop_white^2 + med_hh_income^2+ x_coords + y_coords + x_coords^2 + y_coords^2 | 11 | 3060.93 | 20.99 | 0 | -1519 | 1 |
| PHX_183 | prop_white + med_hh_income + prop_white^2 + med_hh_income^2 + perc_green_tracts^2+ x_coords + y_coords + x_coords^2 + y_coords^2 | 11 | 3061.01 | 21.07 | 0 | -1519 | 1 |
| PHX_152 | perc_green_tracts + prop_white + med_hh_income + perc_green_tracts^2+ x_coords + y_coords + x_coords^2 + y_coords^2 | 10 | 3061.08 | 21.14 | 0 | -1520 | 1 |
| PHX_212 | perc_green_tracts + prop_white x med_hh_income + prop_white^2 + med_hh_income^2 + perc_green_tracts^2+ x_coords + y_coords + x_coords^2 + y_coords^2 | 13 | 3061.19 | 21.25 | 0 | -1517 | 1 |
| PHX_202 | perc_green_tracts + prop_white x med_hh_income + med_hh_income^2 + perc_green_tracts^2+ x_coords + y_coords + x_coords^2 + y_coords^2 | 12 | 3061.36 | 21.42 | 0 | -1519 | 1 |
| PHX_83 | perc_green_tracts x prop_cropland_tracts + prop_white + med_hh_income + med_hh_income^2+ x_coords + y_coords + x_coords^2 + y_coords^2 | 12 | 3061.41 | 21.47 | 0 | -1519 | 1 |
| PHX_114 | perc_green_tracts + prop_white x med_hh_income + prop_white^2 + med_hh_income^2+ x_coords + y_coords + x_coords^2 + y_coords^2 | 12 | 3061.66 | 21.72 | 0 | -1519 | 1 |
| PHX_213 | prop_white x med_hh_income + prop_white^2 + med_hh_income^2 + perc_green_tracts^2+ x_coords + y_coords + x_coords^2 + y_coords^2 | 12 | 3061.76 | 21.82 | 0 | -1519 | 1 |
| PHX_28 | prop_white + med_hh_income + med_hh_income^2+ x_coords + y_coords + x_coords^2 + y_coords^2 | 9 | 3061.97 | 22.03 | 0 | -1522 | 1 |
| PHX_60 | perc_green_tracts + prop_white + med_hh_income+ x_coords + y_coords + x_coords^2 + y_coords^2 | 9 | 3062.08 | 22.15 | 0 | -1522 | 1 |
| PHX_105 | perc_green_tracts + prop_white x med_hh_income + med_hh_income^2+ x_coords + y_coords + x_coords^2 + y_coords^2 | 11 | 3062.09 | 22.16 | 0 | -1520 | 1 |
| PHX_153 | prop_white + med_hh_income + perc_green_tracts^2+ x_coords + y_coords + x_coords^2 + y_coords^2 | 9 | 3062.19 | 22.26 | 0 | -1522 | 1 |
| PHX_203 | prop_white x med_hh_income + med_hh_income^2 + perc_green_tracts^2+ x_coords + y_coords + x_coords^2 + y_coords^2 | 11 | 3062.22 | 22.28 | 0 | -1520 | 1 |
| PHX_145 | perc_green_tracts x prop_desert_tracts + med_hh_income + med_hh_income^2 + perc_green_tracts^2 + prop_desert_tracts^2+ x_coords + y_coords + x_coords^2 + y_coords^2 | 13 | 3062.28 | 22.35 | 0 | -1518 | 1 |
| PHX_59 | prop_desert_tracts x prop_cropland_tracts + med_hh_income + med_hh_income^2+ x_coords + y_coords + x_coords^2 + y_coords^2 | 11 | 3062.77 | 22.83 | 0 | -1520 | 1 |
| PHX_172 | perc_green_tracts + prop_white + med_hh_income + med_hh_income^2 + perc_green_tracts^2+ x_coords + y_coords + x_coords^2 + y_coords^2 | 11 | 3062.88 | 22.95 | 0 | -1520 | 1 |
| PHX_78 | perc_green_tracts + prop_white + med_hh_income + med_hh_income^2+ x_coords + y_coords + x_coords^2 + y_coords^2 | 10 | 3063.9 | 23.96 | 0 | -1522 | 1 |
| PHX_173 | prop_white + med_hh_income + med_hh_income^2 + perc_green_tracts^2+ x_coords + y_coords + x_coords^2 + y_coords^2 | 10 | 3063.99 | 24.06 | 0 | -1522 | 1 |
| PHX_137 | perc_green_tracts + prop_cropland_tracts + prop_white + prop_white^2 + perc_green_tracts^2 + prop_cropland_tracts^2+ x_coords + y_coords + x_coords^2 + y_coords^2 | 12 | 3064.14 | 24.2 | 0 | -1520 | 1 |
| PHX_57 | perc_green_tracts + prop_desert_tracts + prop_cropland_tracts + med_hh_income + med_hh_income^2+ x_coords + y_coords + x_coords^2 + y_coords^2 | 11 | 3064.3 | 24.36 | 0 | -1521 | 1 |
| PHX_58 | perc_green_tracts + prop_desert_tracts x prop_cropland_tracts + med_hh_income + med_hh_income^2+ x_coords + y_coords + x_coords^2 + y_coords^2 | 12 | 3064.8 | 24.87 | 0 | -1520 | 1 |
| PHX_54 | prop_desert_tracts + med_hh_income + med_hh_income^2+ x_coords + y_coords + x_coords^2 + y_coords^2 | 9 | 3064.89 | 24.96 | 0 | -1523 | 1 |
| PHX_53 | perc_green_tracts x prop_desert_tracts + med_hh_income + med_hh_income^2+ x_coords + y_coords + x_coords^2 + y_coords^2 | 11 | 3065.44 | 25.51 | 0 | -1522 | 1 |
| PHX_138 | perc_green_tracts x prop_cropland_tracts + prop_white + prop_white^2 + perc_green_tracts^2 + prop_cropland_tracts^2+ x_coords + y_coords + x_coords^2 + y_coords^2 | 13 | 3065.93 | 25.99 | 0 | -1520 | 1 |
| PHX_149 | perc_green_tracts + prop_desert_tracts + prop_cropland_tracts + med_hh_income + med_hh_income^2 + perc_green_tracts^2 + prop_desert_tracts^2 + prop_cropland_tracts^2+ x_coords + y_coords + x_coords^2 + y_coords^2 | 14 | 3066.14 | 26.21 | 0 | -1519 | 1 |
| PHX_46 | perc_green_tracts + prop_cropland_tracts + prop_white + prop_white^2+ x_coords + y_coords + x_coords^2 + y_coords^2 | 10 | 3066.55 | 26.62 | 0 | -1523 | 1 |
| PHX_127 | perc_green_tracts + prop_cropland_tracts + prop_white + perc_green_tracts^2 + prop_cropland_tracts^2+ x_coords + y_coords + x_coords^2 + y_coords^2 | 11 | 3066.84 | 26.9 | 0 | -1522 | 1 |
| PHX_52 | perc_green_tracts + prop_desert_tracts + med_hh_income + med_hh_income^2+ x_coords + y_coords + x_coords^2 + y_coords^2 | 10 | 3066.91 | 26.97 | 0 | -1523 | 1 |
| PHX_150 | perc_green_tracts + prop_desert_tracts x prop_cropland_tracts + med_hh_income + med_hh_income^2 + perc_green_tracts^2 + prop_desert_tracts^2 + prop_cropland_tracts^2+ x_coords + y_coords + x_coords^2 + y_coords^2 | 15 | 3067.24 | 27.3 | 0 | -1518 | 1 |
| PHX_144 | perc_green_tracts + prop_desert_tracts + med_hh_income + med_hh_income^2 + perc_green_tracts^2 + prop_desert_tracts^2+ x_coords + y_coords + x_coords^2 + y_coords^2 | 12 | 3067.37 | 27.43 | 0 | -1522 | 1 |
| PHX_47 | perc_green_tracts x prop_cropland_tracts + prop_white + prop_white^2+ x_coords + y_coords + x_coords^2 + y_coords^2 | 14 | 3067.85 | 27.91 | 0 | -1523 | 1 |
| PHX_151 | prop_desert_tracts x prop_cropland_tracts + med_hh_income + med_hh_income^2 + perc_green_tracts^2 + prop_desert_tracts^2 + prop_cropland_tracts^2+ x_coords + y_coords + x_coords^2 + y_coords^2 | 11 | 3067.85 | 27.91 | 0 | -1520 | 1 |
| PHX_146 | prop_desert_tracts + med_hh_income + med_hh_income^2 + perc_green_tracts^2 + prop_desert_tracts^2+ x_coords + y_coords + x_coords^2 + y_coords^2 | 11 | 3068.14 | 28.2 | 0 | -1523 | 1 |
| PHX_128 | perc_green_tracts x prop_cropland_tracts + prop_white + perc_green_tracts^2 + prop_cropland_tracts^2+ x_coords + y_coords + x_coords^2 + y_coords^2 | 12 | 3068.65 | 28.71 | 0 | -1522 | 1 |
| PHX_37 | perc_green_tracts + prop_cropland_tracts + prop_white+ x_coords + y_coords + x_coords^2 + y_coords^2 | 9 | 3069.92 | 29.99 | 0 | -1526 | 1 |
| PHX_38 | perc_green_tracts x prop_cropland_tracts + prop_white+ x_coords + y_coords + x_coords^2 + y_coords^2 | 10 | 3071.23 | 31.29 | 0 | -1525 | 1 |
| PHX_24 | med_hh_income+ x_coords + y_coords + x_coords^2 + y_coords^2 | 7 | 3072.66 | 32.72 | 0 | -1529 | 1 |
| PHX_147 | perc_green_tracts + prop_cropland_tracts + med_hh_income + med_hh_income^2 + perc_green_tracts^2 + prop_cropland_tracts^2+ x_coords + y_coords + x_coords^2 + y_coords^2 | 12 | 3073.16 | 33.23 | 0 | -1524 | 1 |
| PHX_55 | perc_green_tracts + prop_cropland_tracts + med_hh_income + med_hh_income^2+ x_coords + y_coords + x_coords^2 + y_coords^2 | 10 | 3073.75 | 33.81 | 0 | -1527 | 1 |
| PHX_25 | med_hh_income + med_hh_income^2+ x_coords + y_coords + x_coords^2 + y_coords^2 | 8 | 3073.89 | 33.96 | 0 | -1529 | 1 |
| PHX_142 | perc_green_tracts + med_hh_income + med_hh_income^2 + perc_green_tracts^2+ x_coords + y_coords + x_coords^2 + y_coords^2 | 10 | 3074.42 | 34.48 | 0 | -1527 | 1 |
| PHX_148 | perc_green_tracts x prop_cropland_tracts + med_hh_income + med_hh_income^2 + perc_green_tracts^2 + prop_cropland_tracts^2+ x_coords + y_coords + x_coords^2 + y_coords^2 | 13 | 3075.14 | 35.21 | 0 | -1524 | 1 |
| PHX_56 | perc_green_tracts x prop_cropland_tracts + med_hh_income + med_hh_income^2+ x_coords + y_coords + x_coords^2 + y_coords^2 | 11 | 3075.45 | 35.52 | 0 | -1527 | 1 |
| PHX_23 | prop_white + prop_white^2+ x_coords + y_coords + x_coords^2 + y_coords^2 | 8 | 3075.51 | 35.57 | 0 | -1530 | 1 |
| PHX_51 | perc_green_tracts + med_hh_income + med_hh_income^2+ x_coords + y_coords + x_coords^2 + y_coords^2 | 9 | 3075.8 | 35.86 | 0 | -1529 | 1 |
| PHX_132 | perc_green_tracts + prop_white + prop_white^2 + perc_green_tracts^2+ x_coords + y_coords + x_coords^2 + y_coords^2 | 10 | 3075.8 | 35.86 | 0 | -1528 | 1 |
| PHX_143 | med_hh_income + med_hh_income^2 + perc_green_tracts^2+ x_coords + y_coords + x_coords^2 + y_coords^2 | 9 | 3075.91 | 35.97 | 0 | -1529 | 1 |
| PHX_12 | prop_desert_tracts x prop_cropland_tracts+ x_coords + y_coords + x_coords^2 + y_coords^2 | 9 | 3076.57 | 36.64 | 0 | -1529 | 1 |
| PHX_19 | perc_green_tracts + prop_desert_tracts + prop_cropland_tracts + perc_green_tracts^2 + prop_desert_tracts^2 + prop_cropland_tracts^2+ x_coords + y_coords + x_coords^2 + y_coords^2 | 12 | 3077.3 | 37.37 | 0 | -1526 | 1 |
| PHX_42 | perc_green_tracts + prop_white + prop_white^2+ x_coords + y_coords + x_coords^2 + y_coords^2 | 9 | 3077.42 | 37.48 | 0 | -1530 | 1 |
| PHX_133 | prop_white + prop_white^2 + perc_green_tracts^2+ x_coords + y_coords + x_coords^2 + y_coords^2 | 9 | 3077.52 | 37.58 | 0 | -1530 | 1 |
| PHX_10 | perc_green_tracts + prop_desert_tracts + prop_cropland_tracts+ x_coords + y_coords + x_coords^2 + y_coords^2 | 9 | 3077.6 | 37.66 | 0 | -1530 | 1 |
| PHX_122 | perc_green_tracts + prop_white + perc_green_tracts^2+ x_coords + y_coords + x_coords^2 + y_coords^2 | 9 | 3077.9 | 37.96 | 0 | -1530 | 1 |
| PHX_22 | prop_white+ x_coords + y_coords + x_coords^2 + y_coords^2 | 7 | 3078.17 | 38.23 | 0 | -1532 | 1 |
| PHX_15 | perc_green_tracts x prop_desert_tracts + perc_green_tracts^2 + prop_desert_tracts^2+ x_coords + y_coords + x_coords^2 + y_coords^2 | 11 | 3078.25 | 38.31 | 0 | -1528 | 1 |
| PHX_11 | perc_green_tracts + prop_desert_tracts x prop_cropland_tracts+ x_coords + y_coords + x_coords^2 + y_coords^2 | 10 | 3078.61 | 38.67 | 0 | -1529 | 1 |
| PHX_20 | perc_green_tracts + prop_desert_tracts x prop_cropland_tracts + perc_green_tracts^2 + prop_desert_tracts^2 + prop_cropland_tracts^2+ x_coords + y_coords + x_coords^2 + y_coords^2 | 13 | 3078.94 | 39.01 | 0 | -1526 | 1 |
| PHX_33 | perc_green_tracts + prop_white+ x_coords + y_coords + x_coords^2 + y_coords^2 | 8 | 3080.05 | 40.12 | 0 | -1532 | 1 |
| PHX_123 | prop_white + perc_green_tracts^2+ x_coords + y_coords + x_coords^2 + y_coords^2 | 8 | 3080.17 | 40.23 | 0 | -1532 | 1 |
| PHX_21 | prop_desert_tracts x prop_cropland_tracts + perc_green_tracts^2 + prop_desert_tracts^2 + prop_cropland_tracts^2+ x_coords + y_coords + x_coords^2 + y_coords^2 | 12 | 3080.34 | 40.4 | 0 | -1528 | 1 |
| PHX_6 | prop_desert_tracts+ x_coords + y_coords + x_coords^2 + y_coords^2 | 7 | 3084.18 | 44.25 | 0 | -1535 | 1 |
| PHX_14 | perc_green_tracts + prop_desert_tracts + perc_green_tracts^2 + prop_desert_tracts^2+ x_coords + y_coords + x_coords^2 + y_coords^2 | 10 | 3084.18 | 44.25 | 0 | -1532 | 1 |
| PHX_5 | perc_green_tracts x prop_desert_tracts+ x_coords + y_coords + x_coords^2 + y_coords^2 | 9 | 3084.54 | 44.61 | 0 | -1533 | 1 |
| PHX_16 | prop_desert_tracts + perc_green_tracts^2 + prop_desert_tracts^2+ x_coords + y_coords + x_coords^2 + y_coords^2 | 9 | 3085.99 | 46.05 | 0 | -1534 | 1 |
| PHX_4 | perc_green_tracts + prop_desert_tracts+ x_coords + y_coords + x_coords^2 + y_coords^2 | 8 | 3086.18 | 46.24 | 0 | -1535 | 1 |
| PHX_17 | perc_green_tracts + prop_cropland_tracts + perc_green_tracts^2 + prop_cropland_tracts^2+ x_coords + y_coords + x_coords^2 + y_coords^2 | 10 | 3091.57 | 51.63 | 0 | -1536 | 1 |
| PHX_7 | prop_cropland_tracts+ x_coords + y_coords + x_coords^2 + y_coords^2 | 7 | 3092.45 | 52.51 | 0 | -1539 | 1 |
| PHX_18 | perc_green_tracts x prop_cropland_tracts + perc_green_tracts^2 + prop_cropland_tracts^2+ x_coords + y_coords + x_coords^2 + y_coords^2 | 11 | 3093.49 | 53.56 | 0 | -1536 | 1 |
| PHX_8 | perc_green_tracts + prop_cropland_tracts+ x_coords + y_coords + x_coords^2 + y_coords^2 | 8 | 3094.36 | 54.42 | 0 | -1539 | 1 |
| PHX_9 | perc_green_tracts x prop_cropland_tracts+ x_coords + y_coords + x_coords^2 + y_coords^2 | 9 | 3095.89 | 55.95 | 0 | -1539 | 1 |
| PHX_13 | perc_green_tracts + perc_green_tracts^2+ x_coords + y_coords + x_coords^2 + y_coords^2 | 8 | 3099.86 | 59.92 | 0 | -1542 | 1 |
| PHX_1 | 1+ x_coords + y_coords + x_coords^2 + y_coords^2 | 6 | 3101.12 | 61.18 | 0 | -1545 | 1 |
| PHX_3 | perc_green_tracts+ x_coords + y_coords + x_coords^2 + y_coords^2 | 7 | 3102.95 | 63.02 | 0 | -1544 | 1 |
